# ε-Caprolactam: MAK-Begründung, Nachtrag

**DOI:** 10.34865/mb10560d9_4ad

**Published:** 2024-12-23

**Authors:** Andrea Hartwig, MAK Commission

**Affiliations:** 1 Institut für Angewandte Biowissenschaften. Abteilung Lebensmittelchemie und Toxikologie. Karlsruher Institut für Technologie (KIT) Adenauerring 20a, Geb. 50.41 76131 Karlsruhe Deutschland; 2 Ständige Senatskommission zur Prüfung gesundheitsschädlicher Arbeitsstoffe. Deutsche Forschungsgemeinschaft, Kennedyallee 40, 53175 Bonn, Deutschland. Weitere Informationen: Ständige Senatskommission zur Prüfung gesundheitsschädlicher Arbeitsstoffe | DFG

**Keywords:** ε-Caprolactam, Reizwirkung, Becherzellhyperplasie, Nase, MAK-Wert, maximale Arbeitsplatzkonzentration, Spitzenbegrenzung, Hautresorption

## Abstract

The German Senate Commission for the Investigation of Health Hazards of Chemical Compounds in the Work Area (MAK Commission) summarized and re-evaluated the data for ε-caprolactam [105-60-2] to derive an occupational exposure limit value (maximum concentration at the workplace, MAK value) considering all toxicological end points. Relevant studies were identified from a literature search. ε-Caprolactam is used mainly for the manufacture of synthetic fibres (especially Nylon 6). In two double-blind studies with male and female volunteers, a NOAEC of 5 mg/m^3^ was derived for chemosensory irritation. In a 13-week inhalation study with whole-body exposure of male and female rats to an aerosol, moderately severe goblet cell hyperplasia was observed in the respiratory mucosa at the lowest concentrations of 24 to 37 mg/m^3^ and above. From the human and animal data, a MAK value of 2 mg/m^3^ was determined. The findings from animal studies suggest that the MAK value derived based on local effects also protects against systemic effects. Peak Limitation Category I with an excursion factor of 2 has been set to prevent chemosensory irritation. In prenatal developmental toxicity studies, ε-caprolactam caused skeletal variations in rats at 500 mg/kg body weight (bw) and day and decreased foetal body weights in rabbits at 150 mg/kg bw and day and above with concurrent maternal toxicity. The NOAELs were 100 and 50 mg/kg bw and day, respectively. The substance is not teratogenic. Damage to the embryo or foetus is unlikely if the MAK value is not exceeded (Pregnancy Risk Group C). ε-Caprolactam is not regarded as genotoxic. The substance did not show carcinogenic potential in studies with rats and mice. Although occupational exposure frequently occurs, only one case of contact sensitization has been reported. Overall, animal studies have not revealed a pronounced sensitizing potential for ε-caprolactam. The substance has been designated with “H” because, according to skin absorption models, skin contact is expected to contribute significantly to systemic toxicity.

**Table:** 

**MAK-Wert (2023)**	**0,42 ml/m^3^ ≙ 2 mg/m^3^ E**
**Spitzenbegrenzung (2002)**	**Kategorie I, Überschreitungsfaktor 2**
	
**Hautresorption (2023)**	**H**
**Sensibilisierende Wirkung**	**–**
**Krebserzeugende Wirkung**	**–**
**Fruchtschädigende Wirkung (1990)**	**Gruppe C**
**Keimzellmutagene Wirkung**	**–**
	
**BAT-Wert**	**–**
	
Synonyma	Aminocaprolactam Aminocapronsäurelactam Caprolactam Cyclohexanonisooxim Epsilon-Caprolactam 2-Oxohexamethylenimin
Chemische Bezeichnung (IUPAC-Name)	Azepan-2-on
CAS-Nr.	105-60-2
Formel	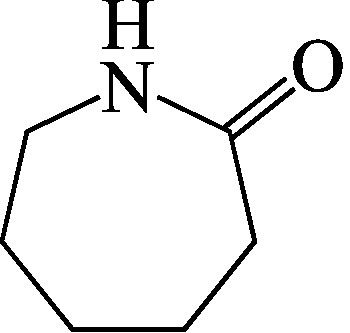
	C_6_H_11_NO
Molmasse	113,16 g/mol
Schmelzpunkt	69,3 °C (ECHA 2022)
Siedepunkt bei 1013 hPa	270,8 °C (ECHA 2022)
Dampfdruck	0,0013 hPa (exp.) bei 20 °C (ECHA 2022) 0,0028 hPa (exp.) bei 26 °C (ECHA 2022)
Dampfsättigung	1,3 ml/m^3^ (6 mg/m^3^) (aus Wert bei 20 °C) 2,8 ml/m^3^ (13 mg/m^3^) (aus Wert bei 26 °C)
log K_OW_	0,12 (exp.) (ECHA 2022)
Löslichkeit	4650 g/l Wasser bei 20 °C (ECHA 2022)
**1 ml/m^3^ (ppm) ≙ 4,695 mg/m^3^**	**1 mg/m^3^ ≙ 0,213 ml/m^3^ (ppm)**

Hinweis: Der Stoff kann gleichzeitig als Dampf und Aerosol vorliegen.

Zu ε-Caprolactam liegen eine Begründung aus dem Jahr 1975 (Henschler 1975), ein Nachtrag von 1990 (Henschler 1990) und ein Nachtrag zur Spitzenbegrenzung von 2002 (Greim 2002) vor. Da neue Studien zu ε-Caprolactam erschienen sind, wird in diesem Nachtrag der MAK-Wert reevaluiert. Zudem wird die Keimzellmutagenität bewertet.

ε-Caprolactam ist das monomere Ausgangsprodukt für die Herstellung von Polymeren, Polyamiden, Thermoplasten und Harzen (ECHA 2022).

Dem SIDS Initial Assessment Report zufolge beträgt die Reinheit von kommerziellem ε-Caprolactam 99,9 % (G/G) (OECD 2001).

ε-Caprolactam ist hygroskopisch und reagiert mit stark oxidierenden Stoffen und starken Basen (NCBI 2021). Daher muss der Stoff unter Luftausschluss gehandhabt werden. Die Herstellung erfolgt im geschlossenen System (ECHA 2022). Der Stoff wird als Schmelze aufbewahrt und transportiert (Ferguson und Wheeler 1973; NCBI 2021).

Aufgrund der physikalischen Eigenschaften (Dampfdruck: 0,0013 hPa bei 20 °C; 0,0028 hPa bei 26 °C (ECHA 2022); Schmelzpunkt: 69,3 °C (ECHA 2022); d. h. bei Raumtemperatur fest) sowie der Herstellungs- und Verarbeitungsprozesse, bei denen bei Erhitzung eine Sublimation sowie bei Abkühlung eine Resublimation auftreten kann, ist am Arbeitsplatz eine Exposition gegen Aerosol und Dampf möglich (ACGIH 2003; OEHHA 2013).

## 1 Allgemeiner Wirkungscharakter

ε-Caprolactam wirkt bei Mensch und Ratte reizend an Auge, Haut und oberem Atemtrakt.

Nach 13-wöchiger Ganzkörperexposition von männlichen und weiblichen Ratten gegen ε-Caprolactam beginnen ab 24 bis 37 mg/m^3^ leichte Effekte mit mittelgradigen Becherzellhyperplasien in der respiratorischen Mucosa der Nase.

In pränatalen Entwicklungstoxizitätsstudien führt der Stoff bei Ratten ab 500 mg/kg KG und Tag und bei Kaninchen ab 150 mg/kg KG und Tag bei gleichzeitiger Maternaltoxizität bei den Feten zu erniedrigten Körpergewichten und verschiedenen skelettalen Variationen. Der Stoff wirkt nicht teratogen.

Eine sensibilisierende Wirkung von ε-Caprolactam an der Haut lässt sich mit der vorhandenen Literatur nicht eindeutig belegen. Zur atemwegssensibilisierenden Wirkung liegen keine positiven Befunde vor.

ε-Caprolactam ist in vitro und in vivo nicht genotoxisch. Der Stoff wirkt bei Ratten und Mäusen nicht kanzerogen.

## 2 Wirkungsmechanismus

Hierzu liegen keine Daten vor.

Der Metabolit 6-Aminohexansäure wird in der Kinderherzchirurgie als Antifibrinolytikum eingesetzt (Sterner 2012). Ein Hinweis auf eine erhöhte Blutgerinnung hat sich aus den Studien mit ε-Caprolactam jedoch nicht ergeben.

## 3 Toxikokinetik und Metabolismus

### 3.1 Aufnahme, Verteilung, Ausscheidung

Männliche F344-Ratten (fünf Tiere/Untersuchungszeitpunkt: 0,5; 1; 2; 3; 4; 6; 15; 24 Stunden) erhielten eine einmalige Gabe per Gavage von 0,18 oder 1,5 mg ^14^C-markiertem ε-Caprolactam/kg KG. Bei der niedrigeren Dosis wurden in 24 Stunden 77,6 % der verabreichten Radioaktivität mit dem Urin, 3,5 % mit den Faeces und 1,5 % mit der Atemluft ausgeschieden (die entsprechenden Werte für die höhere Dosis sind nicht angegeben). Die Exkretion mit dem Urin und der Atemluft war während der ersten sechs Stunden nach der Verabreichung am höchsten. Im Urin wurden nach 24 Stunden nur 2,3 % der Radioaktivität in Form der Ausgangssubstanz detektiert. Die Gabe von 1,5 mg/kg KG führte nach derselben Zeit zu einem höheren Anteil der Ausgangssubstanz im Urin (14,7 %), was eine Limitierung der Metabolisierung anzeigt. Die Eliminationshalbwertszeit der Radioaktivität im Blut lag bei 2,98 Stunden (Unger et al. 1981). Angaben zur Radioaktivität im Gesamtkörper sowie zur Wiederfindung fehlen.

In der Begründung von 1990 ist eine Studie mit Ganzkörperautoradiographie beschrieben. Es wurden männliche sowie weibliche trächtige und nicht trächtige Swiss-Webster-Mäuse einmalig per Schlundsonde oder intravenös mit 6,4 bis 6,9 mg [Carbonyl-^14^C]-Caprolactam/kg KG behandelt und 20 Minuten, drei Stunden und neun Stunden später autoradiographiert. Nach oraler Gabe wurde die Radioaktivität rasch vom Magen resorbiert und über das ganze Tier verteilt, inklusive der Feten. Nach intravenöser Gabe verlief die Verteilung im Vergleich zur oralen Gabe schneller. Es erfolgte eine rasche Ausscheidung mit dem Urin und der Galle. Der über die Galle in den Darm eliminierte Anteil schien nicht über einen enterohepatischen Kreislauf rückresorbiert zu werden. Die Verteilungskinetik war bei männlichen, weiblichen und trächtigen weiblichen Mäusen ähnlich. In den fetalen Geweben wurde keine Radioaktivität retiniert (Henschler 1990; Waddell et al. 1984).

Experimentelle Daten zur dermalen Aufnahme liegen nicht vor. Eine mathematische Abschätzung mittels der Software EPA DERMWIN v2.00 ergab eine Permeabilitätskonstante Kp von 0,000436 cm/h (ECHA 2022).

Der Stoff ist hautreizend. In einem Bühler-Test nach OECD-Prüfrichtlinie 406 wurde als höchste nicht reizende Konzentration 25 % in Wasser ermittelt (ECHA 2022). Für eine 25%ige (nicht mehr reizende) Lösung berechnen sich mit dem Modell von Fiserova-Bergerova et al. (1990) und dem Algorithmus des IH SkinPerm-Modells (Tibaldi et al. 2014) Fluxe von 0,6534 bzw. 0,150 mg/cm^2^ und Stunde. Unter der Annahme einer einstündigen Exposition von 2000 cm^2^ Hautoberfläche würde dies Aufnahmemengen von 1306,8 bzw. 300 mg entsprechen.

### 3.2 Metabolismus

Bei männlichen Sprague-Dawley-Ratten, die zwei bis drei Wochen lang 3 % ε-Caprolactam mit dem Futter bekommen hatten (ca. 3600 mg/kg KG und Tag, Umrechnungsfaktor 0,12 (subakut) nach EFSA Scientific Committee 2012), wurden im Urin vier Ninhydrin-positive Metaboliten gefunden. Davon waren 87,5 % 4-Hydroxycaprolactam und die korrespondierende freie Säure, 8,8 % 6-Aminohexansäure (ε-Aminocapronsäure) und 3,7 % ein nicht identifizierter Metabolit. Im sauren Milieu ergibt sich aus 4-Hydroxycaprolactam ein Gleichgewicht zwischen 6-Amino-γ-caprolacton und 6-Amino-4-hydroxyhexansäure (Kerschner Kirk et al. 1987).

## 4 Erfahrungen beim Menschen

### 4.1 Einmalige Exposition

Seit dem letzten Nachtrag aus dem Jahr 1990 wurden zwei Probandenstudien von derselben Arbeitsgruppe publiziert, die in Tabelle 1 dargestellt sind.

In der ersten Studie wurden je zehn männliche und weibliche Probanden, sechs Stunden lang einmalig gegen 0; 0,15; 0,5 oder 5 mg dampfförmiges ε-Caprolactam/m^3^ exponiert. Alle Probanden waren nach dem Zufallsprinzip und doppelt verblindet an vier aufeinander folgenden Tagen allen Konzentrationen ausgesetzt. Bei der höchsten Konzentration von 5 mg/m^3^ war der Gesamtwert der abgefragten Beschwerden/Symptome statistisch signifikant erhöht. Die Werte bezogen auf den Symptombereich Reizungen (gesamte Reizung, Reizungen der Augen, Reizungen der Nase) waren dagegen nicht statistisch signifikant erhöht. Ab der niedrigsten Konzentration waren die Werte für olfaktorische Symptome konzentrationsabhängig statistisch signifikant erhöht. Die Lidschlussfrequenz, ein objektiver Parameter für die Reizwirkung, wurde durch ε-Caprolactam nicht erhöht. Ophthalmologische Untersuchungen und aktive anteriore Rhinomanometrie waren ohne auffällige Befunde. Daher wurde eine NOAEC für chemosensorische Reizwirkungen von 5 mg/m^3^ abgeleitet (Ziegler et al. 2008).

Eine weitere Untersuchung nach demselben Expositionsschema mit 52 Probanden und einem größeren Untersuchungsumfang ergab keine Auffälligkeiten. Zusätzlich wurde in dieser Studie die Tränenfilmaufrisszeit und die nasale Lavageflüssigkeit untersucht sowie die olfaktorische Funktion genauer betrachtet. Damit wurde die NOAEC für chemosensorische Reizwirkungen von 5 mg/m^3^ bestätigt (Triebig et al. 2016).

Zu den Expositionsbedingungen ist anzumerken, dass in der ersten Studie die Temperatur bei 22 °C und die Luftfeuchtigkeit bei 60 % lag, jeweils ± 10 % (Ziegler et al. 2008). In der zweiten Studie werden für die Expositionskammer Mittelwerte (± SD) für die Temperatur von 28,1 °C (± 2,0) und für die Luftfeuchtigkeit von 63 % (± 5,0) angegeben (Triebig et al. 2016). Bei einer hohen Luftfeuchtigkeit von 70 % und mehr ist eine geringere Akzeptanz des Raumklimas und eine erniedrigte Lidschlussfrequenz zu erwarten (Wolkoff 2018; Wolkoff et al. 2006). Bei der Lidschlussfrequenz lag in der ersten Studie eine hohe Variabilität (0 bis 89 Lidschlüsse/90 s) vor. Die leichte Zunahme der durchschnittlichen Lidschlussfrequenz von 20/90 s vor der Exposition auf 28/90 s bei 5 mg/m^3^ war statistisch nicht signifikant. Aus Abbildung 2 der Publikation ist eine Tendenz zu einer erhöhten Lidschlussfrequenz mit zunehmender Konzentration ersichtlich (Ziegler et al. 2008). Das passt zu einem Anstieg des nasalen Widerstands bei 5 mg/m^3^ und zur subjektiven Symptomatik. In der zweiten Studie mit einer doppelt so hohen Probandenzahl hingegen zeigten sich keine Effekte bei 5 mg/m^3^ (Triebig et al. 2016). In Abbildung 5 der Publikation ist in dieser Studie für die Lidschlussfrequenz weder ein Zeit- noch ein Konzentrationseffekt zu erkennen, d. h. die Ergebnisse der beiden Studien divergieren hinsichtlich dieses Endpunktes. Unter Standardbedingungen liegt die Lidschlussfrequenz bei 15 ± 6/min (ca. 22/90 s). In der ersten Studie ist trotz einer erhöhten Frequenz jedoch keine statistische Signifikanz zur Kontrollgruppe festgestellt worden (Ziegler et al. 2008), was vermutlich auf die geringe Probandenzahl und die hohe Variabilität zurückzuführen ist. In der Nachfolgestudie wurden Probanden mit einer höheren Lidschlussfrequenz als 20 Lidschlüsse/min nicht zuge­lassen und die Probandenzahl war insgesamt größer (Triebig et al. 2016). Zur Generierung und Überwachung der ε-Caprolactamkonzentrationen ist festzustellen, dass in der ersten Studie die Generierung des ε-Caprolactamdampfs mittels Tröpfeln einer wässrigen ε-Caprolactamlösung auf eine erhitzte Glasschale (150 °C) erfolgte (Ziegler et al. 2008). Es stellt sich die Frage, ob bei diesen Temperaturen eventuell noch weitere Verbindungen entstehen können. In der ersten Studie konnte die Konzentrationen die meiste Zeit innerhalb von ± 10 % der Zielkonzentrationen gehal­ten werden (Ziegler et al. 2008). In der zweiten Studie wurden acht 50-min-Luftproben genommen und die Ziel­konzentration wurde mit einer maximalen Abweichung von 8 % erreicht (Triebig et al. 2016). Die Schwankungsbreite der Expositionskonzentrationen ist damit in beiden Studien hoch; bei einem gut funktionierenden Online-Monitoring (Messwerte im Sekundenbereich) liegt die Streuung bei 1 bis 2 %.

**Tab.1:** Wirkungen von ε-Caprolactam nach inhalativer Aufnahme in Probandenstudien

Exposition	Anzahl exponierter Probanden	Konzentration: Befunde	Literatur
0; 0,15; 0,5; 5 mg/m^3^, 6 h, einmalig, Dampf, Ganzkörper, Probanden zufällig u. doppelt-verblindet gegen alle Konzentrationen exponiert	20 gesunde Probanden (10 Männer u. 10 Frauen; 21–38 a)	**ab 0,15 mg/m^3^**: Stärke der Beschwerden/Symptome: SPES-Wert für olfaktorische Symptome ↑ (konzentrationsabhängig); **5 mg/m^3^**: Stärke der Beschwerden/Symptome: SPES-Gesamtwert ↑ (konzentrationsabhängig, keine Adaptation od. Habituation im Verlauf der 6-stündigen Exposition); **5 mg/m^3^**: **NOAEC für chemosensorische Reizwirkungen**; keine Effekte auf/bei: Lidschlussfrequenz, ophthalmologische Untersuchung, aktive anteriore Rhinomanometrie (nasaler Widerstand vor u. nach Exposition); Stärke der Beschwerden/Symptome: SPES-Werte für folgende Endpunkte: gesamte Reizung, Reizungen der Augen, Reizungen der Nase; Persönlichkeitsfaktoren (PANAS) ohne signifikanten Einfluss auf die berichteten Symptome	Ziegler et al. 2008
0; 0,05; 0,5; 5 mg/m^3^, 6 h, einmalig, Dampf, Ganzkörper, Probanden zufällig u. doppelt-verblindet gegen alle Konzentrationen exponiert	52 gesunde Probanden (26 Männer u. 26 Frauen; 19–50 a)	**5 mg/m^3^**: **NOAEC für chemosensorische Reizwirkungen**; keine Effekte auf/bei: Lidschlussfrequenz, Tränenfilmaufrisszeit, Augenrötung, aktive anteriore Rhinomanometrie (nasaler Fluss u. Widerstand), olfaktorische Funktion, nasale Lavage (Gesamtprotein, Interleukin-8; vor, während u. nach der Exposition), Stärke der Beschwerden/Symptome (SPES)	Triebig et al. 2016

a: Jahre; PANAS: Positive And Negative Affect Schedule; SPES: Swedish Performance Evaluation System

Insgesamt genügen beide Studien zwar nicht den Anforderungen an Probandenstudien für chemosensorische Irritationen weil sie nicht den Standardvorgaben (Raumklima: 20 bis 22 °C und 40 bis 60 % Luftfeuchtigkeit, Generierung und Überwachung der Konzentrationen) entsprechen, können jedoch unterstützend für die MAK-Wert-Ableitung herangezogen werden.

### 4.2 Wiederholte Exposition

Bei einer im Nachtrag von 1990 beschriebenen Studie ergaben sich bei den Beschäftigten bis zu ε-Caprolactam­konzentrationen von 33 mg/m^3^ keine Reizwirkungen. Zur Generierung der Luftkonzentration wurde die Abdeckung vom Extruder abgenommen und die Probanden in unterschiedlichen Abständen dazu aufgestellt. An den Plätzen der Probanden erfolgte die Probenahme, vermutlich indem die (heiße?) Luft durch eine Absorptionslösung (­absorption trains) durchgeleitet wurde (Ferguson und Wheeler 1973; Henschler 1990). Hierbei wird die Summe von Dampf und Aerosol erfasst. Die Dampfsättigungskonzentration und der Dampfdruck nehmen mit steigender Temperatur zu. Daher ist unklar, ob nur Dampf oder ein Gemisch aus Dampf und Aerosol vorlag.

Im Nachtrag von 2002 wird eine Arbeitsplatzstudie berichtet, wonach 4,9 mg/m^3^ als Staub nicht zu Beschwerden bei Exponierten führten (Greim 2002; NIOSH 1992).

In einer Arbeitsplatzstudie in den USA wurden 39 männliche Beschäftigte, die zehn oder mehr Jahre in Bereichen mit ε-Caprolactamexposition (21 Beschäftigte aus der Produktionsstätte in Richmond, Virginia und 18 aus der Weiterverarbeitung in Chesterfield, Virginia) gearbeitet hatten, untersucht. Dieselbe Anzahl Beschäftigter ohne ε-Caprolactamexposition wurde hinsichtlich Alter, Ethnie und Rauchen den exponierten Beschäftigten angepasst. Dazu wurden die Daten des medizinischen Zentrums in Chesterfield genutzt, welches für beide Arbeitsstätten zuständig war. Aufnahmekriterien waren neben der Beschäftigungsdauer auch das Vorliegen von Lungenfunktionsuntersuchungen. Luftmessungen zeigten die höchsten durchschnittlichen Konzentrationen in der Betriebsstätte in Chesterfield im Bereich von 4,5 bis 9,9 mg/m^3^. Während kürzerer Expositionszeiten mit Probenahmezeiten von 15 bis 59 Minuten waren die Beschäftigten Konzentrationen von bis zu 34,8 mg/m^3^ ausgesetzt. In der Produktionsstätte in Richmond hingegen lagen die durchschnittlichen Luftkonzentrationen bei 3,7 mg/m^3^ mit kurzzeitigen Expositionen von bis zu 30,8 mg/m^3^. Lungenfunktionsmessungen, inklusive des Verhältnises von Einsekundenkapazität zu forcierter Vitalkapazität (FEV_1_/FVC), ergaben keine Unterschiede zwischen exponierten und nicht exponierten Beschäftigten. Aus den Aufzeichnungen des medizinischen Zentrums im Zeitraum von 1980 bis 1991 geht hervor, dass unter den 878 Konsultationen 30 ε-Caprolactam-Exponierte waren. Bei vier von ihnen konnten die Beschwerden auf ε-Caprolactam zurückgeführt werden. Zwei davon berichteten über Hautreizungen, ein Beschäftigter über Augenreizungen und ein Beschäftigter über die Inhalation von Flusen, die wahrscheinlich aus ε-Caprolactam und teilweise polymerisiertem Nylon bestanden. Die Testsubstanz wurde als gesamtes ε-Caprolactam analysiert ohne zwischen Staub und Dampf zu unterscheiden. Die Unterscheidung Staub/Dampf basierte auf dem Typ des industriellen Prozesses. In Prozessen, bei denen ε-Caprolactam oder das Polymer erhitzt oder angefeuchtet wurde oder in wässriger Lösung vorlag, wurde eine Dampfphase für ε-Caprolactam angenommen. Die ε-Caprolactamreinigung findet ohne Erhitzung statt, während die Produktion von Nylon 6 eine Erhitzung benötigt (Allied-Signal Inc. 1992).

### 4.3 Wirkung auf Haut und Schleimhäute

ε-Caprolactam wirkt beim Menschen reizend an Auge, Haut und oberem Atemtrakt (Henschler 1990).

Neue Untersuchungen liegen nicht vor.

### 4.4 Allergene Wirkung

#### 4.4.1 Hautsensibilisierende Wirkung

Seit dem letzten Nachtrag aus dem Jahr 1990 wurde zur hautsensibilisierenden Wirkung von ε-Caprolactam nur ein Befund im Zusammenhang mit einer beruflichen Exposition publiziert. Ein 62-jähriger Angestellter, der seit 29 Jahren in einer Textilfabrik beschäftigt war und zuletzt 15 Jahre als Leiter der Nylonfaser-Anlage gearbeitet hatte, hatte über 18 Monate ein juckendes, brennendes Ekzem im Gesicht, im Nacken, auf der Brust und an den Gliedmaßen entwickelt. Der Rücken und die Oberarme waren schwächer betroffen. Nach Aussetzen der Arbeit klangen alle Symptome innerhalb von zwei Monaten ab. Zur Überprüfung einer möglichen allergischen Ursache wurden ein offener und ein geschlos­sener Epikutantest durchgeführt. Getestet wurden eine Standard-Kunst- und Klebstoffreihe, ein an der Anlage verwendetes Schmiermittel, Nylon und ε-Caprolactam als Ausgangsmaterial zur Herstellung von Nylon. Beim offenen Test mit ε-Caprolactam (5 % in Wasser) wurde nach einer und zwei Stunden eine einfach positive Reaktion beobachtet, welche sich am 2. Tag nach der Applikation zu einer dreifach positiven Reaktion entwickelt hatte. Der geschlos­sene Epikutantest mit der 5%igen wässrigen Lösung führte zu einer dreifach positiven Reaktion am 2. Tag nach der Applikation. Eine weitere Ablesung zu einem späteren Zeitpunkt erfolgte nicht. Es wurden keine weiteren positiven Reaktionen beobachtet. Auch 15 Kontrollpersonen reagierten nicht auf 5 % ε-Caprolactam (Aguirre et al. 1995).

Des Weiteren liegen zwei Berichte über die vermutete sensibilisierende Wirkung von ε-Caprolactam im Zusammenhang mit chirurgischem Nahtmaterial vor, die im Folgenden beschrieben werden.

Eine als Friseurin tätige Patientin (58 Jahre) entwickelte ekzematöse Läsionen an einer zehn Tage alten Operationswunde, an der chirurgische Fäden gezogen wurden. Die Patientin hatte sich zuvor bereits mehr als 40 chirurgischen Eingriffen zur Entfernung von Muttermalen und zwei Melanomen unterzogen. Ein 48-stündiger Epikutantest mit dem verwendeten Nahtmaterial erzeugte ein Erythem und starkes Brennen, die über eine Woche anhielten. In einem weiteren Epikutantest wurden verschiedene Standardreihen sowie die Bestandteile des Nahtmaterials getestet (5 % ε-Caprolactam und 1 % Azofarbstoff Acid Blue 158, jeweils in Vaseline). Am 3. Tag nach der Applikation wurde eine zweifach positive Reaktion auf ε-Caprolactam und den Azofarbstoff sowie auf Ammoniumpersulfat und 2,5-Diaminotoluol beobachtet. Am 4. Tag wurde eine dreifach positive Reaktion auf den Azofarbstoff beobachtet (k. A. zur Reaktion auf ε-Caprolactam am 4. Tag). In einer weiteren Operation wurde farbloses resorbierbares Nahtmaterial eingesetzt, das von der Patientin gut vertragen wurde. Fünf Kontrollpersonen reagierten negativ auf ε-Caprolactam und den Azofarbstoff Acid Blue 158 (Hausen 2003). In diesem Fall liegt jedoch eine Mischexposition vor und die Informationen zum zeitlichen Reaktionsverlauf sind unvollständig. Ferner lässt sich die Exposition an der nicht intakten Haut nur begrenzt auf die Situation am Arbeitsplatz übertragen.

Bei einer 38-jährigen Patientin mit wiederholten entzündlichen Unverträglichkeitsreaktionen auf ein chirurgisches Nahtmaterial wurde im Epikutantest ein positives Ergebnis auf das betreffende Nahtmaterial beobachtet. Bei der Epikutantestung mit den Bestandteilen des Nahtmaterials wurde unter anderem ε-Caprolactam (k. w. A.) getestet, jedoch hierauf keine Reaktion festgestellt. Als ursächlich wurde schließlich der Azofarbstoff Acid Blue 158 gefunden (Raap et al. 2008).

#### 4.4.2 Atemwegssensibilisierende Wirkung

Es liegen keine Untersuchungen mit Hinweisen auf eine atemwegssensibilisierende Wirkung von ε-Caprolactam vor. Es gibt jedoch einzelne Untersuchungen, bei denen Beschwerden bei Mischexposition auftraten, wobei eine Komponente auch ε-Caprolactam war. Nach Renovierung der Büroräume klagten 32 von 80 Angestellten über Hautprobleme und Rhinitis, die auf neue Teppiche zurückgeführt wurden (Ebbehøj et al. 2015). Einige dieser Teppiche enthielten unter ande­rem auch geringe Mengen ε-Caprolactam (< 0,003 bis 0,29 mg/ml in den aus den Teppichen hergestellten Extrakten), aber Testungen mit dem isolierten ε-Caprolactam erfolgten nicht und die Ursache für die Reaktionen wurde letztlich nicht identifiziert. 

In einer Untersuchung wurden bei 173 Angestellten, die an verschiedenen Teilen einer ε-Caprolactam-Anlage beschäf­tigt waren, spirometrische Untersuchungen durchgeführt. Zur Kontrolle wurden die gleichen Untersuchungen auch an 60 nicht exponierten, gesunden Freiwilligen durchgeführt. Untersucht wurden FVC, FEV_1_, der maximale exspiratorische Spitzenfluss und der maximale exspiratorische Fluss bei 25 und 75 % der forcierten Vitalkapazität. Unter Berücksichtigung von Alter, Größe und Rauchgewohnheiten zeigte die exponierte Gruppe keinen signifikanten Unterschied zur Kontrollgruppe. Laut den Autoren stehen diese Ergebnisse in Einklang mit denen von ande­ren orga­nischen Chemikalien wie Benzol, Cyclohexan, Cyclohexanon, Cyclohexanol, Hydroxylamin und Lactam, die ebenfalls keine chronischen Auswirkungen auf die Lungenfunktionen exponierter Arbeiter haben (Patel 1990). Eine Querschnittsuntersuchung mit alleiniger spirometrischer Untersuchung stellt jedoch keine geeignete Methode dar, um eine Atemwegsallergie zu diagnostizieren oder auszuschließen.

Weitere Untersuchungen liegen nicht vor.

### 4.5 Reproduktionstoxizität

Im SIDS Initial Assessment Report wird über Menstruationsstörungen, vorzeitige Geburten und postnatale Hämorrhagien bei weiblichen Beschäftigten der herstellenden Industrie berichtet. Aufgrund der Mischexposition ist eine Bewertung der reproduktionstoxischen Wirkung von ε-Caprolactam nicht möglich (OECD 2001).

### 4.6 Genotoxizität

Hierzu liegen keine Daten vor.

### 4.7 Kanzerogenität

In einer retrospektiven Kohortenstudie wurde das Leukämierisiko von Beschäftigten in der ε-Caprolactamherstellung, die zudem gegen Benzol exponiert waren, untersucht (Swaen et al. 2005). Aufgrund der Co-Exposition gegen Benzol kann die Studie nicht zur Bewertung der kanzerogenen Wirkung von ε-Caprolactam herangezogen werden.

## 5 Tierexperimentelle Befunde und In-vitro-Untersuchungen

### 5.1 Akute Toxizität

#### 5.1.1 Inhalative Aufnahme

Nach Exposition gegen ein ε-Caprolactam-Aerosol betrug der 4-Stunden-LC_50_-Wert bei männlichen und weiblichen Wistar-Ratten 8160 mg/m^3^ (k. w. A.; ECHA 2022; OECD 2001).

Vier männliche Swiss-Webster-Mäuse wurden 30 Minuten lang gegen 2,2 ml/m^3^, der höchsten möglichen Dampf­konzentration, ganzkörperexponiert. Die Konzentration in der Expositionskammer wurde analytisch bestimmt. Die RD_50_ (Konzentration mit 50%iger Verringerung der Atemfrequenz) lag höher als 2,2 ml/m^3^ (k. w. A.; ECHA 2022).

In einer weiteren Untersuchung wurden vier männliche Mäuse 30 Minuten lang gegen gesättigte Dämpfe von ε-Capro­lactam exponiert (9,4 mg/m^3^). Es wurde eine Abnahme der Atemfrequenz um 8 % festgestellt, die in die erwartete Schwankungsbreite von Kontrolltieren in diesem System fiel. Jedoch wurde bei einem Tier während der Exposition eine leichte sensorische Irritation festgestellt (k. w. A.; ECHA 2022).

#### 5.1.2 Orale Aufnahme

Bei Wistar-Ratten betrug die orale LD_50_ für männliche Tiere 1876 mg/kg KG und für weibliche Tiere 1475 mg/kg KG. Es wurden klonische Konvulsionen beobachtet (k. w. A.; OECD 2001).

#### 5.1.3 Dermale Aufnahme

Bei männlichen und weiblichen Wistar-Ratten lag die dermale LD_50_ bei mehr als 2000 mg/kg KG. Es wurden keine Effekte festgestellt (ECHA 2022; OECD 2001).

#### 5.1.4 Intraperitoneale Aufnahme

Als intraperitoneale LD_50_ für Ratten werden Werte von 800 bzw. 528 mg/kg KG und für Mäuse von 590 bzw. 480 mg/kg KG angegeben (ECHA 2022).

### 5.2 Subakute, subchronische und chronische Toxizität

#### 5.2.1 Inhalative Aufnahme

Seit dem Nachtrag von 1990 ist eine Inhalationsstudie durchgeführt worden. Die histopathologischen Befunde sind in Tabelle 2 dargestellt.

In einer 13-wöchigen Inhalationsstudie ähnlich der OECD-Prüfrichtlinie 413 wurden je zehn männliche und weibliche Sprague-Dawley-Ratten pro Gruppe gegen analytisch bestimmte ε-Caprolactam-Aerosol-Konzentrationen von 0, 24, 70 oder 243 mg/m^3^ sechs Stunden pro Tag, fünf Tage pro Woche ganzkörperexponiert. Zusätzlich gab es weitere zehn Tiere pro Geschlecht und Konzentrationsgruppe, die im Anschluss eine vierwöchige Erholungszeit ohne Exposition hatten. Das Aerosol wurde aus einer 50%igen wässrigen Lösung durch Vernebelung generiert. Die Konzentration in der Luft wurde über Glasfaserfilter gravimetrisch und mittels HPLC bestimmt. Ein etwaiger Dampfanteil wurde nicht erfasst, was zu einer Unterschätzung der Konzentrationen geführt haben könnte. Der MMAD (massenmedianer aerodynamischer Durchmesser) lag bei 2,7 bis 3,2 µm mit einer GSD (geometrische Standardabweichung) von 1,7. Es ist unklar, ob sich der MMAD auf Tröpfchen oder feste Partikel bezieht. Mit Beginn der zweiten Woche wurden während der Zeit in den Expositionskammern in allen Expositionsgruppen behandlungsbedingte erschwerte Atmung und nasaler Ausfluss beobachtet (Huntington Life Sciences 1997; Reinhold et al. 1998). Das OEHHA (California Office of Environmental Health Hazard Assessment) wertete basierend auf den Daten der Originalstudie (Huntington Life Sciences 1997) die beobachteten Symptome aus. Bei 24 mg/m^3^ wiesen 8,1 %, bei 70 mg/m^3^ 12,9 % und bei 243 mg/m^3^ 17 % der Tiere eine erschwerte Atmung vom 6. bis zum 26. Expositionstag auf, während bei den Kontrolltieren keines betroffen war (OEHHA 2013). Diese Effekte sowie feuchte Rasselgeräusche beim Atmen traten auch in den Zeiten ohne Exposition während der Behandlungsperiode auf. Im Allgemeinen war die Inzidenz derartiger Effekte in der zweiten Hälfte der Studie geringer. Bei Kontrolltieren und den Tieren aller Expositionsgruppen wurden histologische Veränderungen in der Nasenschleimhaut wie Hypertrophien/Hyperplasien (nicht getrennt ausgewertet) von Becherzellen der respi­ratorischen Mucosa und intrazytoplasmatisches eosinophiles Material in Epithelzellen der olfaktorischen Mucosa beobachtet. Die Inzidenz derartiger Veränderungen nahm nicht mit der Konzentration zu, jedoch der Schweregrad der Hypertrophien/Hyperplasien von Becherzellen und des intrazytoplasmatischen Materials in Epithelzellen. Am Ende der Erholungszeit waren diese Veränderungen bei den mit ε-Caprolactam behan­delten Tieren nur teilweise zurückgebildet. Ab der niedrigsten Konzentration traten am Larynx squamöse/squamoide Metaplasien/Hyper­plasien (nicht getrennt ausgewertet) des pseudostratifizierten Zylinderepithels auf, das die ventrale seromucöse Drüse abdeckt. Diese Effekte waren in minimaler oder leichter Ausprägung bei männlichen und weiblichen Tieren zu beobachten und waren in der Erholungszeit fast vollständig reversibel. Bei der höchsten Konzentration kam es bei männlichen und weiblichen Tieren zu histologischen Veränderungen in der Mucosa des Larynx (squamöse/squamoide Metaplasie/Hyperplasie mit Keratinisierungen des Epithels), was als adverser Effekt angesehen wurde. Am Ende der vierwöchigen Erholungszeit hatte sich die Keratinisierung im Larynx vollständig zurückgebildet. Die Autoren leiteten eine NOAEC für Reizungen am oberen Atemtrakt von 70 mg/m^3^ ab, weil sie die Befunde bis 70 mg/m^3^ als adaptiv werteten. Bis zur höchsten Konzentration blieben die Untersuchung der motorischen Aktivität und die Functional Observational Battery ohne substanzbedingte Befunde (Huntington Life Sciences 1997; Reinhold et al. 1998).

**Tab.2:** Histopathologische Befunde in der 13-wöchigen Inhalationsstudie an Ratten (Huntington Life Sciences 1997; Reinhold et al. 1998)

	Konzentration (mg/m^3^)							
	♂				♀			
	0	24	70	243	0	24	70	243
**Nase: Befunde nach 13 Wo**								
Anzahl untersuchter Tiere	10	10	10	10	10	10	10	10
**respiratorische Mucosa:**								
**Hypertrophien/Hyperplasien von Becherzellen**	10	10	10	10	9	10	10	10
Minimal	3	4	1	0	2	3	1	0
Leicht	7	4	4	3	7	5	5	5
Mittel	0	2	5	7	0	2	4	5
**olfaktorische Mucosa:**								
**intrazytoplasmatisches eosinophiles Material in Epithelzellen**	7	9	9	10	10	6	9	10
Minimal	7	8	4	3	10	5	6	0
Leicht	0	1	4	2	0	1	2	1
Mittel	0	0	1	3	0	0	1	7
Mittelschwer	0	0	0	2	0	0	0	2
**Nase: Befunde nach 4 Wo Erholung**								
Anzahl untersuchter Tiere	10	10	10	10	10	10	10	10
**respiratorische Mucosa:**								
**Hypertrophien/Hyperplasien von Becherzellen**	10	9	10	10	9	9	8	9
Minimal	5	5	3	1	5	4	1	3
Leicht	5	4	3	5	4	5	5	5
Mittel	0	0	4	4	0	0	2	1
**olfaktorische Mucosa:**								
**intrazytoplasmatisches eosinophiles Material in Epithelzellen**	8	9	9	10	9	10	8	10
Minimal	8	8	6	1	7	9	4	0
Leicht	0	1	1	1	2	1	2	3
Mittel	0	0	2	6	0	0	2	7
Mittelschwer	0	0	0	2	0	0	0	0
**Larynx: Befunde nach 13 Wo**								
Anzahl untersuchter Tiere	10	10	10	10	10	10	10	10
**Mucosa:**								
**squamöse/squamoide Metaplasie/Hyperplasie**	0	3	7	10	0	2	5	10
Minimal	0	3	7	6	0	2	5	6
Leicht	0	0	0	4	0	0	0	4
**metaplastische Keratinisierungen des Epithels**	0	0	0	4	0	0	0	1
Minimal	0	0	0	4	0	0	0	1
**Larynx: Befunde nach 4 Wo Erholung**								
Anzahl untersuchter Tiere	10	10	10	10	10	10	10	10
**Mucosa:**								
**squamöse/squamoide Metaplasie/Hyperplasie**	0	0	0	2	0	0	1	1
Minimal	0	0	0	2	0	0	1	1
**metaplastische Keratinisierungen des Epithels**	0	0	0	0	0	0	0	0

Wo: Wochen

Die Reizwirkung von ε-Caprolactam spiegelt sich als histopathologische Veränderung in den Zielgeweben, den Schleimhäuten von Nase und Larynx, wider. Die minimale squamöse/squamoide Metaplasie/Hyperplasie im Larynx bei 24 und 70 mg/m^3^ ist als adaptive Veränderung und somit nicht als advers zu betrachten. Die bei 243 mg/m^3^ auftretenden Keratinisierungen des Epithels sind aufgrund der Gewebeumbildung als advers anzusehen (Kaufmann et al. 2009). In der olfaktorischen Mucosa der Nase kam es ab 24 mg/m^3^ zu intrazytoplasmatischem eosinophilen Material in Epithelzellen in leichter Ausprägung. Dieser Befund nahm im Schweregrad mit zunehmender Konzentration zu und war nach vierwöchiger Erholungszeit zum Teil nicht reversibel. So verblieb bei 24 mg/m^3^ dieser Befund bei ­jeweils einem männlichen und einem weiblichen Tier nach der Erholungszeit in leichter Ausprägung. Die Kontrolltiere wiesen ebenfalls diesen Befund in minimaler bis leichter Ausprägung auf. Bei „eosinophilem Material“ handelt es sich vermutlich um „hyaline Tropfen“, welche in geringer Zahl bei unbehandelten Ratten und Mäusen auftreten und mit dem Alter zunehmen. Die Anzahl und Größe der Tropfen kann behandlungsbedingt zunehmen, aber die Veränderung ist nicht degenerativ (NTP 2014 b). In der respiratorischen Mucosa der Nase traten ab 24 mg/m^3^ mittelgradige Becherzellhyperplasien/-trophien auf. Mit zunehmender Konzentration nahm der Schweregrad zu und dieser Befund war im mittleren Schweregrad bei 24 mg/m^3^ reversibel, bei den höheren Konzentrationen jedoch nicht. Bei den Kontrolltieren zeigten sich diese Veränderungen ebenfalls in minimaler bis leichter, jedoch nicht in mittelgradiger Ausprägung (Huntington Life Sciences 1997; Reinhold et al. 1998). Bei unbehandelten Nagern enthält das respiratorische Epithel nur wenige Becherzellen (NTP 2014 a). Becherzellhyperplasie und -metaplasie werden häufig in der vorderen Nasenhöhle von Nagern als Antwort auf wiederholte Inhalation von Reizstoffen beobachtet und führen zu vermehrter Schleimbildung (Renne et al. 2009; Rogers 1994). NTP sieht die Becherzellhyperplasie als adap­tive Antwort auf einen Reizstoff an (NTP 2014 a). Bei NTP (2014 a) wird keine Angabe zum Schweregrad der Becherzellhyperplasie vorgenommen. Dieser nimmt bei ε-Caprolactam-Exposition in der 13-Wochen-Studie zu und ist deshalb zusätzlich zu berücksichtigen.

**Ableitung einer LOAEC:** Die Nase ist empfindlicher gegen ε-Caprolactam als der Larynx. Entscheidend für die Ableitung einer NOAEC/LOAEC ist der Befund der mittelgradigen Becherzellhyperplasie im respiratorischen Epithel. Diese wird als Marker für eine subklinische Irritation angesehen, die aber auch zu vermeiden ist, weil auch am Arbeitsplatz eine erhöhte Schleimproduktion in der Nase nicht erwünscht ist (vgl. Begründung zu Methacrylsäure von 2016; Wiench et al. 2016). Daher wird die Konzentration von 24 mg/m^3^ als LOAEC angesehen. Dies wird durch die deutliche Konzentrationsabhängigkeit der Zunahme von Inzidenz und Schweregrad dieses Befundes untermauert. Zudem geht der Befund bei 24 mg/m^3^ mit zeitweilig leicht erhöhtem Nasenausfluss und erschwerter Atmung bei 8 % der Tiere vom 6. bis zum 26. Expositionstag einher (OEHHA 2013). Insgesamt wird dieser Befund bei der Konzentration von 24 mg/m^3^ als leichter, beginnender Effekt gewertet.

**Bemerkungen zur Analytik:** Die Luftkonzentrationen wurden gravimetrisch bestimmt und daher der Dampfanteil nicht erfasst. Theoretisch existiert ε-Caprolactam aufgrund seines Dampfdrucks auch bei hoher Luftfeuchtigkeit in der Dampfphase. Praktisch ist eine messbare Dampfphase für Diethylenglykol, ein Stoff mit ähnlichem Dampfdruck und Siedepunkt, gezeigt worden (Breuer et al. 2015). Es ist davon auszugehen, dass ein Dampfanteil von ε-Caprolactam in der Studie von Reinhold et al. (1998) vorhanden ist. Das OEHHA vertritt die Auffassung, dass bei der angewandten Methode zur Generierung des Aerosols ein etwaiger zusätzlicher Dampfanteil so gering ist, dass dieser unberücksichtigt bleiben kann (OEHHA 2013). Dies wird mit einer Validierungsstudie zur Analytik begründet, in der die Generierung der Testsubstanz mittels einer ähnlichen Methode wie in der Studie von Reinhold et al. (1998)erfolgte (Nau et al. 1984). Zur Probenahme wurde in dieser Studie entweder eine XAD-2-Harzröhre alleine oder ein Glasfaserfilter in Serie vor der XAD-2-Harzröhre eingesetzt. Am Ende der Probenahmestrecke befanden sich zwei Gaswaschflaschen, um aus dem Filter oder der Harzröhre entwichenes ε-Caprolactam zu sammeln. In Abhängigkeit verschiedener ε-Caprolactamkonzentrationen (2,67–4,30; 7,60–13,32; 28,25–39,95 mg/m^3^), Temperatur (19,4 und 35 °C) und Luftfeuchtigkeit (35, 60, 95 %) wurden die ε-Caprolactamkonzentrationen mittels HPLC auf dem Filter, der Harzröhre und in den Gaswaschflaschen untersucht. Bei Expositionsbedingungen, die denen der Studie von Reinhold et al. (1998) (20 bis 27 °C, 21 bis 74 % relative Luftfeuchtigkeit) ähneln, wurde bei einem Einsatz von 2,5- und 10%igen Lösungen ein durchschnittlicher ε-Caprolactamanteil von unter 1 % in der XAD-2-Harzröhre und den Gaswaschflaschen gefun­den, während etwa 99 % des ε-Caprolactams im Filter und der Filterunterstützung gesammelt wurden (Nau et al. 1984). Problematisch an dieser Studie ist, dass die Messwerte bei der Filterunterstützung viel höher sind als beim Filter selbst. Dies ist nur möglich, wenn die Filterunterstützung selbst ε-Caprolactam adsorbiert. Das bedeutet, dass der Dampfanteil viel höher ist, als es die analytische Bestimmung vermuten lässt (Dragan 2022). Bei der gravimetrischen Bestimmung von ε-Caprolactam in der Studie von Reinhold et al. (1998) sind die Filter über Nacht in einem Exsikkator gelagert worden (Konditionierung). Es ist davon auszugehen, dass ein gewisser Anteil von ε-Caprolactam durch Verdunstung vom Filter im Exsikkator verloren gegangen ist und damit unberücksichtigt blieb. Der Anteil an Verdunstungsverlusten im Exsikkator ist nicht abschätzbar, da keine Messwerte dazu vorliegen. Daher sollte die Sättigungskonzentration von 13 mg/m^3^ zu den analytischen Konzentrationen addiert werden, auch um den erwähnten Verdunstungseffekt annähernd auszugleichen (Dragan 2022).

Damit ergeben sich ε-Caprolactam-Konzentrationen von 37, 83 und 256 mg/m^3^. Die tatsächlichen Luftkonzentrationen in der Studie liegen somit zwischen 24 und 37, 70 und 83 und 243 und 256 mg/m^3^.

Die für dieses Tierexperiment generierte Testatmosphäre – durch Lösen des sehr gut wasserlöslichen Stoffs 1:2 in Wasser und Herstellung sehr feiner Tröpfchen mittels Vernebelung ohne Erhitzung – entspricht nicht direkt der am Arbeitsplatz vorliegenden Atmosphäre.

In einer zusätzlichen Studie wurden je vier Meerschweinchen fünf Tage lang, 30 Minuten pro Tag, gegen 0, 3, 10 oder 30 mg ε-Caprolactam/m^3^ als Aerosol ganzkörperexponiert. Die Konzentrationen in der Expositionskammer wurden gravimetrisch bestimmt. Während der Expositionsperiode waren keine Änderungen der Atemfrequenz oder der Atemamplitude festzustellen. Auch die mittels eines Oszillographen erfassten Atemmuster blieben unverändert. Einen Hinweis auf eine Konstriktion der Atemwege gab es nicht (k. w. A.; ECHA 2022).

**Fazit: **In einer 13-wöchigen Inhalationsstudie an männlichen und weiblichen Sprague-Dawley-Ratten mit Ganzkörperexposition gegen ε-Caprolactam-Aerosol wurden bereits ab der niedrigsten Konzentration von 24 bis 37 mg/m^3^ in der Nase mittelgradige Becherzellhyperplasien in der respiratorischen Mucosa und leichtgradiges intrazytoplasmatisches Material in Epithelzellen der olfaktorischen Mucosa sowie im Larynx minimalgradige squamöse/squa­moide Metaplasien/Hyperplasien festgestellt. Nach Ende der vierwöchigen Erholungszeit waren diese Befunde nicht vollständig reversibel (Huntington Life Sciences 1997; Reinhold et al. 1998).

#### 5.2.2 Orale Aufnahme

Vor der 2-Jahre-Kanzerogenitätsstudie ist eine 13-Wochen-Studie durchgeführt worden, die im Nachtrag von 1990 nicht dargestellt ist. In dieser Studie erhielten F344-Ratten (zwölf männliche und zwölf weibliche Tiere/Gruppe) 13 Wochen lang 0, 625, 1250, 2500, 5000 oder 7500 mg/kg Futter (ca. 0, 56, 113, 225, 450, 675 mg/kg KG und Tag, Umrechnungsfaktor 0,09 (subchronisch) nach EFSA Scientific Committee 2012). Bei 675 mg/kg KG und Tag war die Körpergewichtszunahme sowie der Futterverbrauch erniedrigt. Histologische Veränderungen wurden nicht beobachtet (NTP 1982). B6C3F1-Mäuse (zehn männliche und zehn weibliche Tiere/Gruppe) bekamen 0, 5000, 10 000, 15 000, 20 000 oder 30 000 mg/kg Futter (ca. 0, 1000, 2000, 3000, 4000, 6000 mg/kg KG und Tag, Umrechnungsfaktor 0,2 (subchronisch) nach EFSA Scientific Committee 2012). Bei der höchsten Dosis starben zwei weibliche Mäuse. Zudem wiesen männliche und weibliche Tiere ab der niedrigsten Dosis eine erniedrigte Körpergewichtszunahme auf (NTP 1982). Für Ratten lässt sich ein NOAEL für systemische Effekte von 450 mg/kg KG und Tag ableiten. Ein NOAEL für Mäuse konnte nicht abgeleitet werden.

In der nachfolgenden 2-Jahre-Kanzerogenitätsstudie des NTP an männlichen und weiblichen F344-Ratten und B6C3F1-Mäusen wurden bis zu den höchsten Dosen von 375 mg/kg KG und Tag (Ratte) bzw. 2250 mg/kg KG und Tag (Maus), verabreicht mit dem Futter, keine toxischen Effekte festgestellt (Henschler 1990; NTP 1982) (siehe Abschnitt 5.7.2).

Weitere Studien wurden seit dem Nachtrag von 1990 nicht durchgeführt.

**Fazit: **Der NOAEL für systemische Effekte bei Ratten, abgeleitet aus der 2-Jahre-Studie mit Futtergabe, liegt bei 375 mg/kg KG und Tag (Henschler 1990; NTP 1982).

#### 5.2.3 Dermale Aufnahme

Hierzu liegen keine quantitativ bewertbaren Daten vor.

Ein dermaler Expositionsversuch an Meerschweinchen mit 5- und 10%iger wässriger Lösung auf einer Fläche von 4 × 6 cm^2^ mit täglicher Applikation für 62 Tage ergab keine feststellbaren Effekte (ECHA 2022; Hohensee 1951). Die applizierten Volumina sind nicht bekannt, so dass keine quantitative Bewertung möglich ist.

### 5.3 Wirkung auf Haut und Schleimhäute

Hierzu liegen keine validen Daten vor (ECHA 2022; OECD 2001).

Der Stoff ist von den REACH-Registranten als hautreizend (skin irritation 2) und stark augenreizend (eye irritation 2) eingestuft (ECHA 2022).

### 5.4 Allergene Wirkung

#### 5.4.1 Hautsensibilisierende Wirkung

Es liegt ein negativer Bühler-Test nach OECD-Prüfrichtlinie 406 mit 20 weiblichen Hartley-Meerschweinchen vor. Dabei wurden 0,4 ml einer 25%igen wässrigen Lösung von ε-Caprolactam epikutan jeweils dreimal wöchentlich in drei Wochen aufgetragen. Nach 14 Tagen erfolgte die Provokation mit 0,4 ml einer 25%igen wässrigen Lösung, dabei reagierte keines der 20 Tiere nach 24 und 48 Stunden. Laut den Autoren traten minimale Hautirritationen (Grad 0 bis +/–, keine Reaktion bis leichtes unregelmäßiges Erythem) auf. Nach 24 und 48 Stunden wurden Irritationen bei 18 bzw. 4 von 20 behandelten Tieren sowie bei 8 bzw. 2 von 10 Kontrolltieren beobachtet. Als Modifikation der OECD-Prüfrichtlinie 406 erfolgte sieben Tage nach der ersten Provokation eine zweite Provokation an zehn Tieren, dabei zeigte ebenfalls kein Tier eine Reaktion. Die durchschnittlichen Schweregrade in der Behandlungs- und der Kontrollgruppe waren ähnlich. Insgesamt ist das Testergebnis als negativ zu bewerten (ECHA 2022).

In einem Maximierungstest nach OECD-Prüfrichtlinie 406 mit 20 weiblichen Hartley-Meerschweinchen erfolgte ­zunächst eine intradermale Induktion mit 0,1 ml einer 3%igen wässrigen Testsubstanz, gefolgt von einer 24-stündigen epidermalen Applikation von 0,8 ml der 75%igen Testsubstanz am 8. Tag. Die Provokation nach 14 Tagen wurde mit 0,4 ml einer 75%igen (G/V) Lösung der Testsubstanz in Wasser durchgeführt. Nach 24 und 48 Stunden reagierte keines der Tiere positiv. Hautirritationen wurden bei der Testgruppe nach 24 und 48 Stunden jeweils bei 13 bzw. 14 von 20 Tieren (Grad +/–, s. o.) und bei sieben bzw. einem von 20 Tieren (Grad 1, leichtes konfluentes oder mäßig ausgeprägtes unregelmäßiges Erythem) beobachtet. Leichte Ödeme traten nach 24 Stunden bei 7 von 20 Tieren auf, diese waren nach 48 Stunden jedoch abgeklungen. In der Kontrollgruppe wurden nach 24 und 48 Stunden jeweils bei 4 von 5 Tieren fragliche (+/–) und nach 24 Stunden bei einem Tier leichte Hautirritationen (Grad 1) und ein Ödem festgestellt, welche nach 48 Stunden nicht mehr sichtbar waren. Das Testergebnis ist damit negativ (ECHA 2022). 

Im REACH-Registrierungsdossier ist eine weitere, nahezu identische Studie aufgeführt, wobei es Diskrepanzen bei den dokumentierten Ergebnissen gibt. Im zweiten Dokument wurde 48 Stunden nach der Provokation bei 9 von 20 Tieren ein Ödem beobachtet. Hautirritationen wurden bei der Testgruppe nach 24 und 48 Stunden jeweils bei 16 bzw. 18 von 20 Tieren (Grad +/–, s. o.) und bei 4 bzw. 2 von 20 Tieren (Grad 1, s. o.) beobachtet. Höchstwahrscheinlich handelt es sich hierbei jedoch um dieselbe Studie (ECHA 2022). Die Wahl der Konzentrationen und aufgetragenen Volumina ist nicht nachvollziehbar.

Es liegen außerdem einige ältere, nicht nach Prüfrichtlinie durchgeführte und teilweise unvollständig dokumentierte tierexperimentelle Untersuchungen vor, die im Folgenden kurz beschrieben werden.

In einer Untersuchung von 1966 wurde zehn Meerschweinchen einmalig jeweils 0,1 ml einer 1%igen Lösung der Testsubstanz in physiologischer Salzlösung intradermal injiziert. Nach 13 Tagen erfolgte eine offene epikutane Provokation mit 0,4 ml einer 50%igen Lösung in Wasser. Es wurde keine Reaktion beobachtet. Auch bei einer zweiten Provokation mit einer 80%igen Lösung zwölf Tage später wurden keine Irritationen beobachtet. Es liegen keine Informationen zu Kontrolltieren vor (ECHA 2022).

In einer Studie aus dem Jahr 1950 mit zehn Meerschweinchen erfolgte die Induktion durch Applikation einer 66%igen wässrigen Lösung von ε-Caprolactam auf geschädigter Haut bzw. sechs intradermale Injektionen von 0,1 ml einer 0,1%igen wässrigen Lösung. Zwei Wochen nach der Induktion wurde zur Provokation die 66%ige Testsubstanz auf die intakte Haut appliziert. Es wurden mäßige Erytheme bei sieben und schwache Erytheme bei drei von zehn Tieren beobachtet und die Substanz damit als schwach sensibilisierend bewertet (ECHA 2022).

In einem Test aus dem Jahr 1973 wurde bei 30 Meerschweinchen (k. A. zum Stamm) zunächst eine epikutane Induktion mit einer 20%igen wässrigen Lösung durchgeführt. Eine weitere Induktion erfolgte mit einer nicht näher spezifizierten ε-Caprolactam-Oligomer-Lösung. Zur Provokation wurden die gleichen Testzubereitungen in den gleichen Konzentrationen verwendet. Sowohl bei der Induktion mit ε-Caprolactam als auch mit der Oligomer-Lösung wurden nach zehn bzw. nach fünf Tagen irritative Hautreaktionen beobachtet, die reversibel waren. Bei der Provokation mit den beiden Testsubstanzen wurden Erytheme am 5. bis 7. Tag (ε-Caprolactam) und am 2. Tag nach der Applikation (Oligomer) beobachtet. Es liegen keine weiteren Angaben vor. Nach der Provokation war die Histaminbindungsaktivität im Serum < 10 %. Das Testergebnis ist negativ (ECHA 2022). Die Wahl der Konzentrationen für die epikutane Induktion und die Provokation ist nicht nachvollziehbar.

Bei einem Test von 1966 wurde zehn Meerschweinchen (k. A. zum Stamm) zwei Wochen lang an fünf Tagen pro Woche, insgesamt zehnmal, täglich dreimal eine 50%ige Testsubstanz in Ether auf die Flankenhaut aufgetragen. Nach zwei Wochen erfolgte eine erneute Behandlung der anderen Flanke mit der 50%igen Testsubstanz in Ether, wobei nach zwölf Stunden zwar Irritationen, jedoch keine positive allergische Reaktion beobachtet wurde. Bei drei Kontrolltieren wurden ebenfalls nur Irritationen beobachtet. Das Testergebnis ist damit negativ (ECHA 2022).

In einem Testbericht von 1955, bei dem die Effekte der Inhalation von ε-Caprolactam untersucht werden sollten, wurden sechs Meerschweinchen in einer Kammer fünf Stunden pro Tag, insgesamt 67 bis 73 Stunden, gegen den Rauch von ε-Caprolactam exponiert, wobei Konzentrationsangaben fehlen. Dabei wurde die Kondensation der Testsubstanz am Fell der Tiere beobachtet. Daher wurden die Tiere auf eine mögliche Hautsensibilisierung getestet, indem eine epikutane Provokation mit einer 66%igen wässrigen Lösung durchgeführt wurde. Laut den Autoren waren die Tiere sensibilisiert (ECHA 2022). Es fehlen jegliche weitere Angaben.

Ein ähnlicher Test wurde mit vier Hunden durchgeführt, die ebenfalls fünf Stunden pro Tag an insgesamt 43 bis 72 Stunden in einer Kammer gegen den Rauch der Testsubstanz exponiert waren. Eine epikutane Provokation erfolgte mit einer 66%igen wässrigen Lösung von ε-Caprolactam. Laut Angaben der Autoren waren drei der vier Tiere sensibilisiert (ECHA 2022). Jegliche weitere Angaben fehlen.

Bei einem Test aus dem Jahr 1944 wurde 20 Meerschweinchen zur Induktion und Provokation ε-Caprolactam als 200%ige (G/V) wässrige Lösung (2 g Testsubstanz in 1 ml Wasser) offen aufgetragen. Es wurden deutliche Irritationen, jedoch keine Sensibilisierung beobachtet (ECHA 2022).

Aufgrund der nicht den Prüfrichtlinien entsprechenden Durchführungen und der unvollständigen Dokumentationen werden letztere Ergebnisse nicht zur Bewertung herangezogen.

#### 5.4.2 Atemwegssensibilisierende Wirkung

Zur Untersuchung der atemwegssensibilisierenden Wirkung von ε-Caprolactam wurden je vier männliche Hartley-Meerschweinchen an fünf aufeinanderfolgenden Tagen jeweils für 30 Minuten gegen drei Aerosolkonzentrationen (3, 10 oder 30 mg ε-Caprolactam/m^3^) exponiert. Jedes Tier wurde dazu in einen Ganzkörperplethysmographen ­gesetzt, der an eine Primärkammer angeschlossen war, um Druckänderungen durch die Atmungsaktivität der Tiere zu detektieren. Am 19., 26., 33. und 40. Versuchstag wurden die Tiere gegen CO_2_, dann 30 Minuten gegen 30 mg ε-Caprolactam/m^3^, und nochmals 45 Minuten gegen CO_2_ exponiert. Nach der fünftägigen Induktionsbehandlung zeigte kein Tier eine signifikante Wirkung auf das respiratorische System (Untersuchungsmethode nicht spezifiziert) und auch nach den Auslösebehandlungen wurde keine unmittelbare Reaktion festgestellt. Nach der Provokation wurden die Lungen entnommen und untersucht (k. w. A.). Die Autoren bewerten das Ergebnis als negativ (ECHA 2022). Da viele Angaben fehlen, kann die Untersuchung nicht zur Bewertung herangezogen werden.

### 5.5 Reproduktionstoxizität

Zu diesem Endpunkt liegen keine neuen Untersuchungen vor.

#### 5.5.1 Fertilität

In der bereits im Nachtrag von 1990 beschriebenen 3-Generationen-Studie an zehn männlichen und 20 weiblichen F344-Ratten wurde den Tieren 0, 1000, 5000 oder 10 000 mg ε-Caprolactam/kg Futter verabreicht (ca. 0, 90, 450, 900 mg/kg KG und Tag, Umrechnungsfaktor 0,09 (subchronisch) nach EFSA Scientific Committee 2012). Die Dosierungszeit betrug zehn Wochen. Danach wurden die Tiere verpaart und die nachfolgenden Generationen wurden jeweils nach einer 10-wöchigen Wachstumsphase nach dem gleichen Schema behandelt. Bis zur höchsten Dosis wurden keine Effekte auf die Fertilität und die Reproduktionsorgane beobachtet. Bei der höchsten Dosis kam es bei den männlichen und weiblichen Elterntieren (1. Generation) sowie den Jungtieren der 2. Generation zu erniedrigten Körpergewichtszunahmen und Futteraufnahmen. In der 3. Generation verstärkte sich dieser Effekt (Henschler 1990; Serota et al. 1988). Der NOAEL für Fertilität liegt bei 900 mg/kg KG und Tag und der NOAEL für Parentaltoxizität bei 450 mg/kg KG und Tag. Ein NOAEL für Perinataltoxizität wird nicht abgeleitet, da die Tiere nicht während der Trächtigkeit behandelt wurden.

#### 5.5.2 Entwicklungstoxizität

Die Entwicklungstoxizitätsstudien zu ε-Caprolactam sind in Tabelle 3 aufgeführt.

In einer pränatalen Entwicklungstoxizitätsstudie an F344-Ratten wurden bei den Muttertieren ab der niedrigsten Dosis von 100 mg/kg KG und Tag Urinverfärbungen, raues Fell, roter Ausfluss aus der Vagina, blutige Krusten um Augen, Maul und Nase sowie Hockstellung beobachtet. Die Feten wiesen ab 500 mg/kg KG und Tag eine erhöhte Inzidenz an skelettalen Variationen auf. Es wurde keine substanzbedingte Teratogenität festgestellt (Gad et al. 1987; Henschler 1990). Der NOAEL für Entwicklungstoxizität liegt bei 100 mg/kg KG und Tag. Ein NOAEL für Maternaltoxizität kann nicht abgeleitet werden.

In einer weiteren pränatalen Entwicklungstoxizitätsstudie an Neuseeländer-Kaninchen aus dem gleichen Prüflabor wurde ab 150 mg/kg KG und Tag maternale Toxizität in Form von erniedrigter Körpergewichtszunahme festgestellt. Bei den Feten wurde ab dieser Dosis ein erniedrigtes Körpergewicht beobachtet. ε-Caprolactam führte nicht zu terato­genen Effekten (Gad et al. 1987; Henschler 1990). Der NOAEL für Entwicklungs- und Maternaltoxizität liegt bei 50 mg/kg KG und Tag.

**Tab.3:** Entwicklungstoxizitätsstudien nach Verabreichung von ε-Caprolactam

Spezies, Stamm, Anzahl pro Gruppe	Exposition	Befunde	Literatur
**Ratte**, F344, 20 ♀	**GD 6–15**, 0, 100, 500, 1000 mg/kg KG u. d, Schlundsonde, Reinheit > 98 %, Vehikel: destilliertes Wasser, Untersuchung GD 20, **ähnlich der OECD TG 414**	**kein NOAEL Maternaltoxizität**; **ab 100 mg /kg KG**: Muttertiere: Urinverfärbungen, raues Fell, roter Ausfluss aus der Vagina, blutige Krusten um Augen, Maul u. Nase, Hockstellung; **100 mg/kg KG**: **NOAEL Entwicklungstoxizität**; **ab 500 mg/kg KG**: Muttertiere: KG-Zunahme ↓ (GD 6–11), Futteraufnahme ↓, Feten: skelettale Variationen ↑ (unvollständige Ossifikationen u. zusätzliche Rippen, Kontrolle: 6,3 %, 100 mg/kg KG: 5,8 %, 500 mg/kg KG: 12,2 %, 1000 mg/kg KG: 53,6 %); **bei 1000 mg/kg KG**: Muttertiere: Mortalität ↑ (9 Tiere), KG ↓ (GD 15: 10 %, GD 20: 11 %, Anzahl Resorptionen ↑ (41,3 %, Kontrolle: 4,6 %); keine Teratogenität	Gad et al. 1987; Henschler 1990
**Kaninchen**, Neuseeländer, 25 ♀	**GD 6–28**, 0, 50, 150, 250 mg/kg KG u. d, Schlundsonde, Reinheit > 98 %, Vehikel: destilliertes Wasser, Untersuchung GD 29, **ähnlich der OECD TG 414**	**50 mg/kg KG**: **NOAEL Entwicklungs- u. Maternaltoxizität**; **ab 150 mg/kg KG**: Muttertiere: KG-Zunahme ↓ (GD 6–9, GD 9–12); Feten: KG ↓ (15 %, 250 mg/kg KG: 12 %); **bei 250 mg/kg KG**: Muttertiere: Mortalität ↑ (4 Tiere); Feten: Inzidenz uni- od. bilaterale 13. Rippe ↑; Trächtigkeitsrate in allen Gruppen mindestens 80 %, keine Teratogenität	Gad et al. 1987; Henschler 1990

d: Tag; GD: Gestationstag; PND: Postnataltag; TG: Test Guideline (Prüfrichtlinie)

Eine Studie mit inhalativer Exposition von Ratten (Henschler 1990; Khadzhieva 1969) wird aufgrund der mangelhaften Dokumentation von Methoden und Ergebnissen nicht zur Bewertung herangezogen.

### 5.6 Genotoxizität

Daten zur Genotoxizität sind bereits im Nachtrag von 1990 aufgeführt (Henschler 1990). Zudem liegen ausführliche Zusammenfassungen der Daten zur Genotoxizität vor (Ashby und Shelby 1989; IARC 1999). Eine ausführliche Darstellung der Studien ist in den Tabellen 4 und 5 zu finden.

#### 5.6.1 In vitro

In zahlreichen Tests an Bakterien zeigte ε-Caprolactam keine mutagenen Effekte (Greene et al. 1979; Henschler 1990; IARC 1999). Eine Inhibierung der DNA-Synthese wurde in HeLa S3-Zellen bis 200 mM nicht festgestellt (Heil und Reifferscheid 1992). Es wird über mehrere Untersuchungen berichtet, in denen ε-Caprolactam nicht zur Induktion der DNA-Reparatursynthese in Rattenhepatozyten führte (IARC 1999). Die Substanz löste in mehreren Tests an verschie­denen Säugetierzellen keinen Schwesterchromatidaustausch aus (Henschler 1990; IARC 1999; Norppa und Järventaus 1989). ε-Capro­lactam hatte in einer Vielzahl von Untersuchungen an Säugetierzellen keine klastogenen Effekte zur Folge (IARC 1999). Jedoch liegen mehrere positive Chromosomenaberrationstests bei sehr hohen Konzentrationen nahe oder bei Zytotoxizität vor (Henschler 1990; Kristiansen und Scott 1989; Norppa und Järventaus 1989; Sheldon 1989 b) bzw. ohne genaue Angaben zur Zytotoxizität (Henschler 1990; Howard et al. 1985). In einer dieser Untersuchungen kam es neben der Induktion struktureller Chromosomenaberrationen auch zur Induktion von Polyploidien (Norppa und Järventaus 1989). Der OECD-Prüfrichtlinie 473 zufolge sollte – falls keine Präzipitatbildung oder limitierende Zytotoxizität auftritt – die höchste Testkonzentration 10 mM oder 2 mg/ml betragen, je nachdem, welche die niedrigere Konzentration ist. Damit liegen die eingesetzten Testkonzentrationen aller drei Tests weit über der empfohlenen höchsten Konzentration nach Prüfrichtlinie. Daher wird ε-Caprolactam als nicht klastogen in vitro angesehen.

Auch in verschiedenen Säugetierzellen induzierte ε-Caprolactam keine mutagenen Effekte (Greene et al. 1979; Henschler 1990; IARC 1999).

**Tab.4:** Genotoxizität von ε-Caprolactam in vitro

Endpunkt	Testsystem	Konzentration	Zytotoxizität	Ergebnis		Literatur
				–m. A.	+m. A.	
Genmutation	S. typhimurium TA98, TA100, TA1535, TA1537, TA1538	0, 5000, 10 770, 23 210, 50 000 µg/Platte	k. A.	–	–	Greene et al. 1979; Henschler 1990
Genmutation mehrere Untersuchungen	S. typhimurium TM677, TA97, TA98, TA100, TA102, TA1535, TA1537, TA1538	0–25 000 µg/Platte	k. A.	–	–	IARC 1999
Inhibierung DNA-Synthese	HeLa S3-Zellen	0–200 mM	k. A.	–	n. d.	Heil und Reifferscheid 1992
UDS mehrere Untersuchungen	Rattenhepatozyten	0–1000 µg/ml	k. A.	–	n. d.	IARC 1999
SCE	CHO-Zellen	0, 1765, 3542, 7073, 14 145, 28 290 µg/ml	–m. A.: ab 14 145 µg/ml; +m. A.: bei 28 290 µg/ml	–	14 145 µg/ml: +	Henschler 1990; Norppa und Järventaus 1989
SCE mehrere Untersuchungen	CHO-Zellen, V79-Zellen, RL4-Zellen, Humanlymphozyten	0–17 000 µg/ml	k. A.	–	–	IARC 1999
CA	Humanlymphozyten	0, 2500, 5000, 7500 µg/ml	bei 10 000 µg/ml: MI: 25 % der Kontrolle	n. d.	7500 µg/ml: +^a)^	Henschler 1990; Kristiansen und Scott 1989
	Humanlymphozyten	–m. A.: 0, 1415, 2829, 5658, 11 316 µg/ml; +m. A.: 0, 2829, 5658, 11 316, 22 632, 45 264 µg/ml	^b)^ –m. A.: bei 11 316 µg/ml; +m. A.: bei 45 264 µg/ml	ab 2829 µg/ml: + Polyploidie 5658 µg/ml: +^a)^ CA	ab 11 316 µg/ml: + Polyploidie 22 632 µg/ml: +^a)^ CA	Henschler 1990; Norppa und Järventaus 1989
	Humanlymphozyten	0, 270, 1370, 2750 µg/ml	2750 µg/ml: MI 50–80 % ↓ (k. w. A.)	270 µg/ml: +	270 µg/ml: +	Henschler 1990; Howard et al. 1985
	Humanfibroblasten	0, 550, 2750, 5500 µg/ml	5500 µg/ml: MI 50–80 % ↓	5500 µg/ml: +^a)^	5500 µg/ml: +^a)^	Henschler 1990; Sheldon 1989 b
	CH1-L-Zellen	0, 200–2000 µg/ml		–	n. d.	Danford 1985
CA mehrere Untersuchungen	CHO-Zellen, CHL-Zellen, RL4-Zellen	0–17 000 µg/ml	k. A.	–	–	IARC 1999
MN	CHO-Zellen	0–113 µg/ml	k. A.	–	–	IARC 1999
Genmutation HPRT	CHO-Zellen	0, 2222, 3333, 5000, 7500, 11 250 µg/ml	ab 7500 µg/ml	–	–	Greene et al. 1979; Henschler 1990
Genmutation HPRT, TK, Ouabainresistenz mehrere Untersuchungen	CHO-Zellen, V79-Zellen, Mauslymphomzellen L5178Y, BALB/c-3T3, Humanlymphozyten	0–15 000 µg/ml	k. A.	–	–	IARC 1999

a) bei hohen Konzentrationen nahe oder bei Zytotoxizität

b) k. A. zur Testsubstanz, nur allgemeine Aussage zum Zytotoxizitätstest bei 10 getesteten Stoffen, Zytotoizität geringer als 50 %

–: negatives Ergebnis; +: positives Ergebnis; CA: Chromosomenaberrationen, HPRT: Hypoxanthin-Guanin-Phosphoribosyltransferase; k. A.: keine Angabe; m. A.: metabolische Aktivierung; MI: mitotischer Index; MN: Mikronuklei; n. d.: nicht durchgeführt; SCE: Schwesterchromatidaustausch; TK: Thymidin-Kinase; UDS: autoradiographischer DNA-Reparatursynthesetest

#### 5.6.2 In vivo

In zahlreichen In-vivo-Untersuchungen wirkte ε-Caprolactam nicht klastogen (siehe Tabelle 5).

An der Leber von CD-1-Swiss-Mäusen und F344-Ratten führte die Substanz zu DNA-Schäden, die mittels der alka­lischen Elution erfasst wurden. Dies wurde von den Autoren ohne weitere Begründung als Veränderung der Chromatinkonformation interpretiert. Weitere Tests an der Mäuseleber mit zwei anderen Techniken (fluorometrisch und viskometrisch bestimmte alkalische Entwindung) ergaben keine DNA-Schäden (Parodi et al. 1989). Es ist technisch nicht möglich, mittels der alkalischen Elution Chromatinkonformationen zu detektieren. Da die Dosierungen nahe der intraperitonealen LD_50_ bei Mäusen von 480 bis 590 mg/kg KG (ECHA 2022; Parodi et al. 1989) lagen, ist das Ergebnis zudem als fraglich anzusehen.

An Drosophila (Henschler 1990; IARC 1999; Vogel 1989) und in Fellfleckentests an den Mäusestämmen (C57BL × T)F1 und (T × HT)F1 (Fahrig 1989; Fahrig und Neuhäuser-Klaus 1989; Henschler 1990; Neuhäuser-Klaus und Lehmacher 1989) wurden ε-Caprolactam-induzierte mitotische Rekombinationen in Somazellen beobachtet. Im Fellfleckentest der einen Studie ergab sich bei einer der beiden 500-mg/kg-Gruppen eine statistisch signifikante Erhöhung der Häufigkeit der SGR (Spots of Genetic Relevance), bei 400 mg/kg KG nicht (Fahrig 1989; Fahrig und Neuhäuser-Klaus 1989). In der zweiten Studie wurde nur in einer von vier 500-mg/kg-Gruppen eine statistisch signifikante Erhöhung der SGR festgestellt, bei 700 mg/kg KG jedoch nicht (Henschler 1990; Neuhäuser-Klaus und Lehmacher 1989). Somit liegt keine Dosis-Wirkungs-Beziehung vor. Die maximale Erhöhung der mitotischen Rekombinationen erreichte knapp das Doppelte. Auf die in beiden Studien verwendeten hohen Dosen nahe der intraperitonealen LD_50_ bei Mäusen von 480 bis 590 mg/kg KG (ECHA 2022; Parodi et al. 1989) ist ebenfalls hinzuweisen. Daher könnten die Effekte auch durch Toxizität bedingt sein.

Die im Fellfleckentest erfassten reziproken Rekombinationen in Somazellen werden durch mitotisches Crossover verursacht und gleichzeitig sind in diesem Test keine Mutationen beobachtet worden (Fahrig 1993). Im Nachtrag von 1990 wird erläutert, dass die Bedeutung der mitotischen Rekombinationen für die Krebsentstehung ungeklärt ist (Henschler 1990). Diese Ansicht wird auch im SIDS Initial Assessment Report von der OECD vertreten (OECD 2001). Hervorzuheben ist, dass ε-Caprolactam in Kanzerogenitätsstudien an Ratten und Mäusen nicht kanzerogen wirkte (Henschler 1990; NTP 1982).

In zwei Untersuchungen an Keimzellen, UDS-Test (autoradiographischer DNA-Reparatursynthesetest) und Spermienkopfanomalie-Test, wurden keine genotoxischen Effekte durch ε-Caprolactam festgestellt (Henschler 1990; Salamone 1989; Working 1989). Der letztere ist nur dazu geeignet, die Erreichbarkeit der Keimzellen zu zeigen (ICPEMC 1983; Salamone et al. 1985; Wild 1984).

**Tab.5:** Genotoxizität von ε-Caprolactam in vivo

Testsystem		Exposition	Ergebnis	Zytotox./Anmerkungen	Literatur
**Somazellen**					
SMART	Drosophila, > 2000 Augen/Dosis untersucht	bis 4 d, 0; 2,5–20 mM, Nährlösung, untersucht nach 10–11 d	–		Henschler 1990; Vogel 1989
SMART	Drosophila, k. w. A.	0, 565 µg/ml, Nährlösung, k. w. A.	–		IARC 1999
SMART	Drosophila, k. w. A.	5000 µg/ml, Nährlösung, k. w. A.	–		IARC 1999
SMART	Drosophila, k. w. A.	45 000 µg/ml, Nährlösung, k. w. A.	+		IARC 1999
SMART	Drosophila, k. w. A.	1000 µg/ml, Nährlösung, k. w. A.	+		IARC 1999
SLRL	Drosophila, > 300 Flügel/Dosis untersucht	bis 4 d, 0–20 mM, Nährlösung, untersucht nach 10–11 d	♂: –, ♀: +	keine Zunahme mit steigender Dosis	Henschler 1990; Vogel 1989
SLRL	Drosophila, k. w. A.	15 000 µg/ml, Injektion, k. w. A.	(+)		IARC 1999
SCE Knochenmark	Maus, B6C3F1, 4 ♂/Dosis	einmalig, 0, 175, 350, 700 mg/kg KG, i.p., Lösungsmittel: Maiskeimöl, untersucht nach 24 h	–	höchste Dosis: 50 % der MTD, Positivkontrolle: DMBA	Henschler 1990; McFee und Lowe 1989
DNA-Schäden (Comet-Assay) Hepatozyten	Ratte, F344, 3 ♂/Dosis	einmalig, 0, 750 mg/kg KG, Gavage, Lösungsmittel: Wasser, untersucht nach 12, 24, 48 h	–	Positivkontrolle: DMNA	Bermudez et al. 1989; Henschler 1990
DNA-Schäden (Comet-Assay) Hepatozyten	Ratte, Sprague Dawley, k. w. A.	zweimal, 0, 425 mg/kg KG u. d, oral, k. w. A.	–		IARC 1999
DNA-Schäden (alkalische Elution) Leber	Maus, CD1-Swiss, 2 (4 h) u. 5 (24 h) ♂/Dosis, Kontrolle: 8 ♂	einmalig, 0, 580 mg/kg KG (≈i.p. LD_50_), i.p., Lösungsmittel: Wasser, untersucht nach 4, 24 h	(+)	Positivkontrolle: DMNA	Parodi et al. 1989
DNA-Schäden (alkalische Elution) Leber	Ratte, F344, 3–4 ♂/Dosis	einmalig, 0, 580 mg/kg KG, i.p., Lösungsmittel: Wasser, untersucht nach 4 h	(+)	Positivkontrolle: DMNA	Parodi et al. 1989
DNA-Schäden (alkalische Entwindung: fluorometrisch u. viskometrisch) Leber	Ratte, F344, 3–4 ♂/Dosis	einmalig, 0, 580 mg/kg KG, i.p., Lösungsmittel: Wasser, untersucht nach 24 h	–	Positivkontrolle: DMNA	Parodi et al. 1989
UDS Hepatozyten	Ratte, F344, 3 ♂/Zeitpunkt	einmalig, 0, 750 mg/kg KG, Gavage, Lösungsmittel: Wasser, untersucht nach 12, 24, 48 h	–	Positivkontrolle: DMNA	Bermudez et al. 1989; Henschler 1990
CA Knochenmark	Maus, 1C3F1, je 5 ♂ u. ♀/Zeitpunkt	einmalig, 0, 1000 mg/kg KG, Gavage, Lösungsmittel: destilliertes Wasser, untersucht nach 24, 30, 48 h	–	nach 30 h: mitotischer Index ↓, Positivkontrolle: Benzo[a]pyren	Adler und Ingwersen 1989; Henschler 1990
CA Knochenmark	Maus, B6C3F1, 8 ♂/Dosis	einmalig, 0, 175, 350, 700 mg/kg KG, i.p., Lösungsmittel: Maiskeimöl, untersucht nach 18 h	–	höchste Dosis: 50 % der MTD, Positivkontrolle: DMBA	Henschler 1990; McFee und Lowe 1989
MN Knochenmark	Maus, k. A. zum Stamm, je 5 ♂ u. ♀/Zeitpunkt	einmalig, 0, 435, 700 mg/kg KG, Gavage, Lösungsmittel: Olivenöl, untersucht nach 24, 36, 48 h	–	bei 700 mg/kg KG: MN leicht ↑ (auch im Wiederholungsexperiment), aber im Bereich der historischen Kontrolle, Prozentsatz PCE unverändert, Positivkontrolle: Cyclophosphamid	Henschler 1990; Sheldon 1989 a
MN Knochenmark	Maus, ICR/JCL, 5 ♂/Dosis	einmalig, 0, 125, 250, 500, 1000 mg/kg KG, i.p., Lösungsmittel: physiologische Kochsalzlösung, untersucht nach 24, 36, 48 h	–	PCE/NCE unverändert, Positivkontrolle: Mitomycin C	Henschler 1990; Ishidate und Odagiri 1989
Fellfleckentest	Maus, (T × HT)F1, 500 mg/kg KG: 4 Gruppen zu je 27–46 ♀ u. 16–22 ♀ Kontrollen, 700 mg/kg KG: 23 ♀ u. 18 ♀ Kontrollen	einmalig an GD 9, 0, 500, 700 mg/kg KG, i.p., Lösungsmittel: Phosphat-gepufferte Kochsalzlösung	+	keine Embryotoxizität, bei 500 mg/kg KG: nur in 1 von 4 Gruppen: statistisch signifikante Erhöhung der SGR, kein signifikanter Unterschied bei 700 mg/kg KG → keine DWB; Interpretation als Ergebnis mitotischer Rekombination (Fahrig und Neuhäuser-Klaus 1989)	Henschler 1990; Neuhäuser-Klaus und Lehmacher 1989
Fellfleckentest	Maus, (C57BL × T)F1, 400 mg/kg KG: 96 ♀, 500 mg/kg KG: 2 Gruppen zu 95 bzw. 116 ♀ u. 75 ♀ Kontrollen	einmalig an GD 9, 0, 400, 500 mg/kg KG, i.p., Lösungsmittel: Phosphatpuffer	+	Embryotoxizität: Wurfgröße ↓, abnormale Morphologie ↑, bei 1/2 Gruppen bei 500 mg/kg KG: statistisch signifikante Erhöhung der SGR (Fahrig 1989; Fahrig und Neuhäuser-Klaus 1989); Interpretation als Ergebnis mitotischer Rekombination (Fahrig 1989; Fahrig und Neuhäuser-Klaus 1989)	Fahrig 1989; Henschler 1990
**Keimzellen**					
UDS Spermatozyten	Ratte, F344, 3 ♂/Zeitpunkt	einmalig, 0, 750 mg/kg KG, Gavage, Lösungsmittel: Wasser, untersucht nach 12, 24, 48 h	–	Positivkontrolle: Cyclophosphamid	Henschler 1990; Working 1989
Spermienkopfanomalien Spermien	Maus, B6C3F1, 6–10 ♂/Dosis	5 d, 0, 222, 333, 500, 750, 1125 mg/kg KG u. d, Gavage, Lösungsmittel: Maiskeimöl, untersucht nach 24 h	–	Toxizität ab 500 mg/kg KG: mindestens ein totes Tier/Dosis, Positivkontrolle: DMBA	Henschler 1990; Salamone 1989

–: negatives Ergebnis; +: positives Ergebnis; (+): Ergebnis nicht eindeutig positiv; CA: Chromosomenaberrationen; d: Tag; DMBA: 7,12-Dimethylbenz[a]anthracen; DMNA: Dimethylnitrosamin; DWB: Dosis-Wirkungs-Beziehung; GD: Gestationstag; i.p.: intraperitoneal; k. (w.) A.: keine (weiteren) Angaben; MN: Mikronuklei; MTD: maximal tolerierbare Dosis; NCE: normochromatische Erythrozyten; PCE: polychromatische Erythrozyten; SCE: Schwesterchromatidaustausch; SGR: Spots of Genetic Relevance; SLRL: Sex-Linked Recessive Lethal Test; SMART: Somatic Mutation and Recombination Test; UDS: autoradiographischer DNA-Reparatursynthesetest; Zytotox.: Zytotoxizität

#### 5.6.3 Fazit

ε-Caprolactam wird als nicht genotoxisch in vitro und in vivo angesehen (Henschler 1990; IARC 1999; OECD 2001).

### 5.7 Kanzerogenität

#### 5.7.1 Kurzzeitstudien

ε-Caprolactam war im SA7-Virus-Transformationstest und im Transformationstest an sekundären Hamsterembryo-Zellen unwirksam (Greene et al. 1979; Henschler 1990).

Drei Transformationstests an BALB/c-3T3-, C3H 10T½- und Embryonalzellen des Syrischen Hamsters verliefen marginal positiv, während die Ergebnisse von viral verstärkten Zelltransformationstests negativ waren (IARC 1999).

Je 15 männlichen, sechs Wochen alten F344/DuCrj-Ratten wurden zuerst einmalig intraperitoneal 100 mg N-Nitroso­diethylamin/kg KG verabreicht, anschließend zwei Wochen lang viermal pro Woche intraperitoneal 20 mg N-Methyl-N-nitrosoharnstoff/kg KG und Tag und danach zwei Wochen lang 0,1 % N-Bis(2-hydroxypropyl)nitrosamin im Trink­wasser. Danach erhielten die Tiere 16 Wochen lang 10 000 mg ε-Caprolactam/kg Futter (ca. 900 mg/kg KG und Tag, Umrechnungsfaktor 0,09 (subchronisch) nach EFSA Scientific Committee 2012). Dreißig Ratten bekamen nach dem ersten Behandlungsschritt unbehandeltes Futter und dienten als Kontrollen. Zusätzlich wurde fünf Ratten das Vehikel ohne die kanzerogenen Stoffe verabreicht und anschließend wurden die Tiere mit ε-Caprolactam im Futter behandelt. Bei den ε-Caprolactam-behandelten Tieren wurden bei histologischen Untersuchungen von Leber, Niere, Milz und Schilddrüse sowie bei der Quantifizierung von Glutathion-S-Transferase (plazentale Form) (GST-P)-positiven Foci in der Leber keine auffälligen Befunde erhoben (Fukushima et al. 1991).

In einer weiteren Kurzzeitstudie wurde 14 sechs Wochen alten männlichen F344-Ratten einmalig intraperitoneal 200 mg N-Nitrosodiethylamin/kg KG injiziert. Nach zwei Wochen bekamen die Tiere sechs Wochen lang entweder 10 000 mg ε-Caprolactam/kg Futter (ca. 900 mg/kg KG und Tag, Umrechnungsfaktor 0,09 (subchronisch) nach EFSA Scientific Committee 2012) oder unbehandeltes Futter. In der dritten Woche wurden die Tiere einer 2/3-Hepatektomie unterzogen. Bei den ε-Caprolactam-behandelten Tieren wurde keine statistisch signifikante Erhöhung der Anzahl GST-P-positiver Foci in der Leber im Vergleich zu den Kontrollen festgestellt (Hasegawa und Ito 1992).

#### 5.7.2 Langzeitstudien

Hierzu liegen keine neuen Daten vor.

In einer 2-Jahre-Kanzerogenitätsstudie des NTP an je 50 männlichen und weiblichen F344-Ratten und B6C3F1-Mäusen erhielten die Ratten 0, 3750 oder 7500 mg ε-Caprolactam/kg Futter und die Mäuse 0, 7500 oder 15 000 mg ε-Caprolactam/kg Futter. Dies entspricht für die Ratten 0; 187,5 oder 375 mg/kg KG und Tag und für die Mäuse 0, 1125 oder 2250 mg/kg KG und Tag unter Verwendung eines Umrechnungsfaktors von 0,05 (Ratte) bzw. 0,15 (Maus) (chronisch) (EFSA Scientific Committee 2012). Die Substanz erwies sich bei beiden Spezies nicht als kanzerogen oder toxisch (Henschler 1990; NTP 1982).

Die IARC (International Agency for Reseach on Cancer) bewertete ε-Caprolactam im Jahr 1999 als wahrscheinlich nicht kanzerogen für den Menschen (Gruppe 4) (IARC 1999). In einer Reevaluierung ihrer fünf Kanzerogenitäts-Kategorien (Gruppen 1, 2A, 2B, 3, 4) stellte die IARC im Jahr 2019 fest, dass sich nur eine einzige Substanz, ε-Caprolactam, in Gruppe 4 befand. Es wird erläutert, dass die Substanz 1982 das erste Mal bewertet und danach noch dreimal reevaluiert (1986, 1987, 1999) worden ist (Smith und Perfetti 2019). Im Jahr 2019 wurden die beiden Gruppen 3 und 4 zur Gruppe 3 zusammengefügt (Samet et al. 2020). Daher befindet sich ε-Caprolactam bei IARC nun in Gruppe 3 (not classifiable as to its carcinogenicity to humans).

## 6 Bewertung

Kritische Effekte von ε-Caprolactam sind beim Menschen die chemosensorische Reizwirkung und bei der Ratte die lokale Reizwirkung nach Inhalation.

**MAK-Wert. **In Arbeitsplatzstudien wurden bei ε-Caprolactamkonzentrationen von 4,9 mg/m^3^ (NIOSH 1992), 4,5 bis 9,9 mg/m^3^ (Allied-Signal Inc. 1992) und 33 mg/m^3^ (Ferguson und Wheeler 1973) bei den Beschäftigten keine Reizwirkungen beschrieben.

In zwei Probandenstudien an Männern und Frauen (insgesamt 72 Personen mit einmaliger Exposition über je einen Zeitraum von sechs Stunden) hatte dampfförmiges ε-Caprolactam bis zur jeweils höchsten Konzentration von 5 mg/m^3^ keine chemosensorischen Reizeffekte zur Folge (Triebig et al. 2016; Ziegler et al. 2008). Beide Studien genügen zwar nicht völlig den Anforderungen an Probandenstudien für sensorische Irritation weil sie nicht den Standardvorgaben (Raumklima: 20 bis 22 °C und 40 bis 60 % Luftfeuchtigkeit) entsprechen, können jedoch unterstützend für die MAK-Wert-Ableitung herangezogen werden.

In einer 13-wöchigen Inhalationsstudie an männlichen und weiblichen Sprague-Dawley-Ratten mit Ganzkörper­exposi­tion gegen Aerosol/Dampf sind ab der niedrigsten Konzentration von 24 bis 37 mg/m^3^ mittelgradige Becherzellhyper­plasien in der respiratorischen Mucosa und leichtgradiges intrazytoplasmatisches Material in Epithelzellen der olfak­torischen Mucosa der Nase sowie minimalgradige squamöse/squamoide Metaplasien/Hyperplasien im Larynx festgestellt worden, die am Ende der vierwöchigen Erholungszeit nicht vollständig reversibel gewesen sind (Huntington Life Sciences 1997; Reinhold et al. 1998). Entscheidend für die Ableitung einer NOAEC/LOAEC ist der Befund „mittelgradige Becherzellhyperplasie im respiratorischen Epithel“. Die Becherzellhyperplasie im respiratorischen Epithel wird als Marker für eine subklinische Irritation angesehen, die aber auch zu vermeiden ist, weil auch am Arbeitsplatz eine erhöhte Schleimproduktion in der Nase nicht erwünscht ist (vgl. Begründung zu Methacrylsäure von 2016; Wiench et al. 2016). Zudem treten bei 24 bis 37 mg/m^3^ zeitweilig leicht erhöhter Nasenausfluss und erschwerte Atmung bei 8 % der Tiere vom 6. bis zum 26. Expositionstag auf (OEHHA 2013). Daher wird die Konzentration von 24 bis 37 mg/m^3^ als LOAEC mit leichten, beginnenden Effekten gewertet (siehe Abschnitt 5.2.1).

Ausgehend von der **LOAEC im Bereich von 24 bis 37 mg/m^3^** ergibt sich unter der Berücksichtigung der Extrapolation LOAEC/NAEC (1:3), der Speziesextrapolation für Effekte an der Nase (1:3) und der Zeitextrapolation (1:2) (Brüning et al. 2014) eine Luftkonzentration von 1,3 bis 2,1 mg/m^3^. Mit dem Preferred Value Approach ergibt sich damit ein MAK-Wert von 1 mg/m^3^ bis 2 mg/m^3^. Dieser Wert steht im Widerspruch zu den Arbeitsplatzerfahrungen mit Exposition gegen Dampf und Aerosol. So sind bei 4,9 mg/m^3^ (NIOSH 1992), 4,5 bis 9,9 mg/m^3^ (Allied-Signal Inc. 1992) und 33 mg/m^3^ (Ferguson und Wheeler 1973) keine Reizeffekte festgestellt worden.

Bei einer **Plausibilitätsbetrachtung** unter der Berücksichtigung folgender Aspekte lässt sich ein MAK-Wert von 2 mg/m^3^ ableiten:

- Ein **MAK-Wert von 2 mg/m^3^** wird der Probandenstudie mit den Kritikpunkten bezüglich der Standardvorgaben, der Generierung und der Überwachung der Substanzkonzentrationen gerecht. Auch die Kritikpunkte an der Tierstudie, wie die unterschiedliche Testatmosphäre, die Generierung der Testsubstanz in Wasser sowie die Analytik ohne Erfassung des Dampfes, werden ausreichend berücksichtigt.
- Da ε-Caprolactam gut wasserlöslich ist, ist anzunehmen, dass der erste Wirkort des Dampfes das Auge und nicht die Nase ist.
- Das Aerosol wirkt auf Larynx/Nase, der Dampf nur auf das Auge (bei geringer Luftfeuchte), weil keine Nasen-/Racheneffekte an Probanden auftreten.
- Am Arbeitsplatz erfolgt die Handhabung des Stoffes als Schmelze, so dass eine Exposition im Dampfbereich nahe der Sublimation auftritt.
- Die Probandenstudien mit einer NOAEC für chemosensorische Reizwirkungen von 5 mg/m^3^ und die Arbeitsplatz­erfahrungen sind höher zu werten als der Tierversuch.

Der NOAEL für systemische Effekte bei Ratten, abgeleitet aus der 2-Jahre-Fütterungsstudie, liegt bei 375 mg/kg KG und Tag (Henschler 1990; NTP 1982). Zur toxikokinetischen Übertragung in eine Konzentration in der Luft am Arbeitsplatz wird berücksichtigt: die tägliche Exposition der Tiere im Vergleich zur fünftägigen Exposition pro Woche am Arbeitsplatz (7:5), der dem toxikokinetischen Unterschied zwischen Ratte und dem Menschen entsprechende speziesspezifische Korrekturwert (1:4), die orale Resorption von 79,1 % (experimentelle Bestimmung bei Ratten: Unger et al. 1981), das Körpergewicht (70 kg) und das Atemvolumen (10 m^3^) des Menschen, die angenommene 100%ige inhalative Resorption sowie die Übertragung der Tierversuchsdaten auf den Menschen (1:2). Damit errechnet sich eine Konzentration von 363 mg/m^3^, die weitaus höher ist als der abgeleitete MAK-Wert von 2 mg/m^3^.

**Spitzenbegrenzung. **Da die Reizwirkung im Vordergrund steht, bleibt ε-Caprolactam weiterhin der Kurzzeitwert-Kategorie I zugeordnet. Der Überschreitungsfaktor wird auf 2 festgesetzt, weil die NOAEC für chemosensorische Reizwirkungen aus den Probandenstudien bei 5 mg/m^3^ liegt.

**Fruchtschädigende Wirkung. **In einer pränatalen Entwicklungstoxizitätsstudie mit Schlundsondengabe an F344-Ratten wurde bei den Feten bei 500 mg ε-Caprolactam/kg KG und Tag eine erhöhte Inzidenz an skelettalen Variationen bei gleichzeitiger Maternaltoxizität festgestellt. Der NOAEL für Entwicklungstoxizität liegt bei 100 mg/kg KG und Tag (Gad et al. 1987; Henschler 1990). Bei Neuseeländer-Kaninchen führte der Stoff im gleichen Studientyp mit Schlundsondengabe ab 150 mg/kg KG und Tag zu erniedrigtem Körpergewicht der Feten, ebenfalls bei gleichzeitiger Maternaltoxizität (ernie­drigte Körpergewichtszunahme). Der NOAEL für Entwicklungstoxizität liegt bei 50 mg/kg KG und Tag (Gad et al. 1987; Henschler 1990). Bei beiden Spezies wirkte ε-Caprolactam in diesen Studien nicht teratogen.

Zur toxikokinetischen Übertragung der NOAEL für pränatale Entwicklungstoxizität von 100 mg/kg KG und Tag (Ratte) und 50 mg/kg KG und Tag (Kaninchen) in eine Konzentration in der Luft am Arbeitsplatz werden berücksichtigt: die den toxikokinetischen Unterschieden zwischen Ratte bzw. Kaninchen und dem Menschen entsprechenden speziesspezifischen Korrekturwerte (1:4 bzw. 1:2,4), die orale Resorption von 79,1 % (experimentelle Bestimmung bei Ratten: Unger et al. 1981, die auch für Kaninchen angenommen wird), das Körpergewicht (70 kg) und das Atemvolumen (10 m^3^) des Menschen sowie die angenommene 100%ige inhalative Resorption. Damit errechnen sich entsprechende Konzentrationen von 138 mg/m^3^ bzw. 115 mg/m^3^. Die 69- bzw. 58-fachen Abstände zum MAK-Wert von 2 mg/m^3^ sind ausreichend groß. Zudem hat sich ε-Caprolactam bei Ratten und Kaninchen als nicht teratogen gezeigt. Daher wird die Zuordnung zur Schwangerschaftsgruppe C bestätigt.

**Krebserzeugende Wirkung. **ε-Caprolactam erwies sich in Kanzerogenitätsstudien des NTP an Ratten und Mäusen als nicht kanzerogen (Henschler 1990; NTP 1982). Der Stoff wird daher weiterhin nicht in eine Kategorie für krebserzeugende Arbeitsstoffe eingestuft.

**Keimzellmutagene Wirkung. **ε-Caprolactam wird als nicht genotoxisch in vitro und in vivo angesehen. Daher erfolgt keine Einstufung in eine Kategorie für Keimzellmutagene.

**Hautresorption. **Zur perkutanen Resorption liegen keine experimentellen Untersuchungen vor. Der dermale LD_50_-Wert für eine akute Exposition bei Ratten lag bei mehr als 2000 mg/kg KG (keine Effekte festgestellt). Zur wiederholten dermalen Exposition liegen keine quantitativ bewertbaren Daten vor. Der Stoff ist hautreizend. In einem Bühler-Test nach OECD-Prüfrichtlinie 406 wurde als höchste nicht reizende Konzentration 25 % in Wasser ermittelt (ECHA 2022).

Für den Menschen lässt sich aus den Modellrechnungen (Abschnitt 3.1) eine maximale dermale Aufnahme von 300 mg (IH SkinPerm-Modell von Tibaldi et al. 2014) bzw. 1306,8 mg (Modell von Fiserova-Bergerova et al. 1990) bei Exposition gegen eine 25%ige (nicht mehr reizende) Lösung unter Standardbedingungen (2000 cm^2^ Hautoberfläche, eine Stunde Exposition) abschätzen.

Aus der berechneten systemischen NAEC für den Menschen von 363 mg/m^3^ (siehe MAK-Wert) und dem Atemvolumen von 10 m^3^ in acht Stunden bei angenommener 100%iger inhalativer Resorption lässt sich eine systemisch tolerable Menge von 3630 mg ableiten. 

Da die aus den Modellen abgeschätzte dermale Aufnahme zum Teil mehr als 25 % dieser systemisch noch tolerablen Menge beträgt, sind systemische Effekte bei Hautkontakt auch bei Einhaltung des MAK-Wertes nicht sicher auszuschließen. ε-Caprolactam wird daher mit „H“ markiert.

**Sensibilisierende Wirkung. **Trotz weiter Anwendung von ε-Caprolactam als Monomer in der Polyamidsynthese liegt nur ein Befund zur kontaktsensibilisierenden Wirkung beim Menschen vor. Tierexperimentelle Untersuchungen lieferten in der Gesamtschau überwiegend negative Ergebnisse. Insgesamt kommt ε-Caprolactam keine ausgeprägte kontaktallergene Wirkung zu. Positive Befunde zur atemwegssensibilisierenden Wirkung liegen nicht vor. ε-Caprolactam wird daher weder mit „Sh“ noch mit „Sa“ markiert.

## References

[id_DUK_557] American Conference of Governmental Industrial Hygienists ACGIH (2003). Caprolactam.

[id_DUK_558] Adler I. D., Ingwersen I. (1989). Evaluation of chromosomal aberrations in bone marrow of 1C3F1 mice. Mutat Res.

[id_DUK_559] Aguirre A., González Pérez R., Zubizarreta J., Landa N., Sanz de Galdeano C., Díaz Pérez J. L. (1995). Allergic contact dermatitis from epsilon-caprolactam. Contact Dermatitis.

[id_DUK_560] Allied-Signal Inc. (1992). Caprolactum: a study of current workers with cover letter dated 090392..

[id_DUK_561] Ashby J., Shelby M. D. (1989). Overview of the genetic toxicity of caprolactam and benzoin. Mutat Res.

[id_DUK_562] Bermudez E., Smith-Oliver T., Delehanty L. L. (1989). The induction of DNA-strand breaks and unscheduled DNA synthesis in F-344 rat hepatocytes following in vivo administration of caprolactam or benzoin. Mutat Res.

[id_DUK_563] Breuer Dietmar, Dragan George C., Friedrich Claudia, Möhlmann Carsten, Zimmermann Ralf (2015). Development and field testing of a miniaturized sampling system for simultaneous sampling of vapours and droplets. Environ Sci Process Impacts.

[id_DUK_564] Brüning Thomas, Bartsch Rüdiger, Bolt Hermann Maximillian, Desel Herbert, Drexler Hans, Gundert-Remy Ursula, Hartwig Andrea, Jäckh Rudolf, Leibold Edgar, Pallapies Dirk, Rettenmeier Albert W., Schlüter Gerhard, Stropp Gisela, Sucker Kirsten, Triebig Gerhard, Westphal Götz, van Thriel Christoph (2014). Sensory irritation as a basis for setting occupational exposure limits. Arch Toxicol.

[id_DUK_565] Danford N, Ashby J., de Serres F. J., Draper M., Ishidate M, Margolin B. H., Matter B. E., Shelby M. D. (1985). Evaluation of short-term tests for carcinogens: report of the International Programme on Chemical Safety’s collaborative study on in vitro assays.

[id_DUK_566] Dragan C.-G. (2022). email to:.

[id_DUK_567] Ebbehøj Niels E., Agner Tove, Zimerson Erik, Bruze Magnus (2015). Outbreak of eczema and rhinitis in a group of office workers in Greenland. Int J Circumpolar Health.

[id_DUK_568] (2022). ε-Caprolactam (CAS Number 105-60-2). Registration dossier. Joint submission, first publication 03 Mar 2011, last modification 19 Apr 2023.

[id_DUK_569] EFSA Scientific Committee (2012). Guidance on selected default values to be used by the EFSA Scientific Committee, Scientific Panels and Units in the absence of actual measured data. EFSA J.

[id_DUK_570] Fahrig R. (1989). Possible recombinogenic effect of caprolactam in the mammalian spot test. Mutat Res.

[id_DUK_571] Fahrig R., Fahrig R. (1993). Mutationsforschung und genetische Toxikologie..

[id_DUK_572] Fahrig R., Neuhäuser-Klaus A. (1989). Positive effect of caprolactam in the mammalian spot test: an overview. Mutat Res.

[id_DUK_573] Ferguson W. S., Wheeler D. D. (1973). Caprolactam vapor exposures. Am Ind Hyg Assoc J.

[id_DUK_574] Fiserova-Bergerova V., Pierce J. T., Droz P. O. (1990). Dermal absorption potential of industrial chemicals: criteria for skin notation. Am J Ind Med.

[id_DUK_575] Fukushima S., Hagiwara A., Hirose M., Yamaguchi S., Tiwawech D., Ito N. (1991). Modifying effects of various chemicals on preneoplastic and neoplastic lesion development in a wide-spectrum organ carcinogenesis model using F344 rats. Jpn J Cancer Res.

[id_DUK_576] Gad S. C., Robinson K., Serota D. G., Colpean B. R. (1987). Developmental toxicity studies of caprolactam in the rat and rabbit. J Appl Toxicol.

[id_DUK_577] Greene Elliott J., Friedman Marvin A., Sherrod Julie A. (1979). In vitro mutagenicity and cell transformation screening of caprolactam. Environ Mutagen.

[id_DUK_578] Greim H. (2002). Gesundheitsschädliche Arbeitsstoffe, Toxikologisch-arbeitsmedizinische Begründung von MAK-Werten.

[id_DUK_579] Hasegawa R., Ito N. (1992). Liver medium-term bioassay in rats for screening of carcinogens and modifying factors in hepatocarcinogenesis. Food Chem Toxicol.

[id_DUK_580] Hausen Bjoern M. (2003). Allergic contact dermatitis from colored surgical suture material: contact allergy to epsilon-caprolactam and acid blue 158. Am J Contact Dermat.

[id_DUK_581] Heil J., Reifferscheid G. (1992). Detection of mammalian carcinogens with an immunological DNA synthesis-inhibition test. Carcinogenesis.

[id_DUK_582] Henschler D. (1975). Gesundheitsschädliche Arbeitsstoffe, Toxikologisch-arbeitsmedizinische Begründung von MAK-Werten.

[id_DUK_583] Henschler D. (1990). Gesundheitsschädliche Arbeitsstoffe, Toxikologisch-arbeitsmedizinische Begründung von MAK-Werten.

[id_DUK_584] Hohensee F. (1951). On the pharmacological and physiological effects of ε-caprolactam. Faserforsch. Textiltechn..

[id_DUK_585] Howard C. A., Sheldon T, Richardson C. R., Ashby J., de Serres F. J., Draper M., Ishidate M, Margolin B. H., Matter B. E., Shelby M. D. (1985). Evaluation of short-term tests for carcinogens: report of the International Programme on Chemical Safety’s collaborative study on in vitro assays.

[id_DUK_586] Huntington Life Sciences (1997). 13-week inhalation toxicity study (with a 4-week recovery) of caprolactam (494-95A) in the rat via whole-body exposure with cover letter dated 04/30/1997.

[id_DUK_587] International Agency for Research on Cancer IARC (1999). Re-evaluation of some organic chemicals, hydrazine and hydrogen peroxide.

[id_DUK_588] International Commission for Protection Against Environmental Mutagens and Carcinogens ICPEMC (1983). Committee 1 final report: screening strategy for chemicals that are potential germ-cell mutagens in mammals. Mutat Res.

[id_DUK_589] Ishidate M, Odagiri Y. (1989). Negative micronucleus tests on caprolactam and benzoin in ICR/JCL male mice. Mutat Res.

[id_DUK_590] Kaufmann Wolfgang, Bader Rainer, Ernst Heinrich, Harada Takanori, Hardisty Jerry, Kittel Birgit, Kolling Angelika, Pino Michael, Renne Roger, Rittinghausen Susanne, Schulte Agnes, Wöhrmann Thomas, Rosenbruch Martin (2009). 1st International ESTP Expert Workshop: “Larynx squamous metaplasia”. A re-consideration of morphology and diagnostic approaches in rodent studies and its relevance for human risk assessment. Exp Toxicol Pathol.

[id_DUK_591] Kerschner Kirk L., Lewis B. A., Ross D. A., Morrison M. A. (1987). Identification of ninhydrin-positive caprolactam metabolites in the rat. Food Chem Toxicol.

[id_DUK_592] Khadzhieva E. D. (1969). [The effect of caprolactam on the reproductive function of white rats]. Gig Sanit.

[id_DUK_593] Kristiansen E., Scott D. (1989). Chromosomal analyses of human lymphocytes exposed in vitro to caprolactam. Mutat Res.

[id_DUK_594] McFee A. F., Lowe K. W. (1989). Caprolactam and benzoin: tests for induction of chromosome aberrations and SCEs in mouse bone marrow. Mutat Res.

[id_DUK_595] Nau D.R., Darr R.W., Gad S.C., Pai S.V. (1984). Validation study of a method for monitoring personnel exposure to caprolactam.

[id_DUK_596] (2021). Caprolactam. PubChem compound summary for CID 7768.

[id_DUK_597] Neuhäuser-Klaus A., Lehmacher W. (1989). The mutagenic effect of caprolactam in the spot test with (T × HT) F_ 1_ mouse embryos. Mutat Res.

[id_DUK_598] The National Institute for Occupational Safety and Health NIOSH (1992). Health hazard evaluation report No 90-174.

[id_DUK_599] Norppa H., Järventaus H. (1989). Induction of chromosome aberrations and sister-chromatid exchanges by caprolactam in vitro. Mutat Res.

[id_DUK_600] National Toxicology Program NTP (1982). NTP Technical report on the carcinogenesis bioassay of caprolactam (CAS No. 105-60-2) in F344 rats and B6C3F1 mice (feed study)..

[id_DUK_601] (2014). Respiratory system. Nose – hyperplasia, goblet cell. Nonneoplastic lesion atlas.

[id_DUK_602] (2014). Respiratory system. Nose, epithelium – accumulation, hyaline droplet. Nonneoplastic lesion atlas.

[id_DUK_603] Organisation of Economic Co-operation and Development OECD (2001). Caprolactum. SIDS Initial Assessment Report for 12^th^ SIAM.

[id_DUK_604] (2013). Caprolactam (aerosol, vapor & particulate). Adopted reference exposure levels for caprolactam.

[id_DUK_605] Parodi S., Abelmoschi M. L., Balbi C., De Angeli M. T., Pala M., Russo P., Taningher M., Santi L. (1989). DNA damage in mouse and rat liver by caprolactam and benzoin, evaluated with three different methods. Mutat Res.

[id_DUK_606] Patel M.B. (1990). Study of lung functions in caprolactam workers. Indian J Ind Med.

[id_DUK_607] Raap Ulrike, Wieczorek Dorothea, Kapp Alexander, Wedi Bettina (2008). Allergic contact dermatitis to acid blue 158 in suture material. Contact Dermatitis.

[id_DUK_609] Reinhold R. W., Hoffman G. M., Bolte H. F., Rinehart W. E., Rusch G. M., Parod R. J., Kayser M. (1998). Subchronic inhalation toxicity study of caprolactam (with a 4-week recovery) in the rat via whole-body exposures. Toxicol Sci.

[id_DUK_610] Renne Roger, Brix Amy, Harkema Jack, Herbert Ron, Kittel Birgit, Lewis David, March Thomas, Nagano Kasuke, Pino Michael, Rittinghausen Susanne, Rosenbruch Martin, Tellier Pierre, Wohrmann Thomas (2009). Proliferative and nonproliferative lesions of the rat and mouse respiratory tract. Toxicol Pathol.

[id_DUK_611] Rogers D. F. (1994). Airway goblet cells: responsive and adaptable front-line defenders. Eur Respir J.

[id_DUK_612] Salamone M. F. (1989). Abnormal sperm assay tests on benzoin and caprolactam. Mutat Res.

[id_DUK_613] Salamone M.F., Ashby J, de Serres F.J., Shelby M.D., Margolin B.H., Ishidate M., Becking C.G. (1985). Evaluation of short-term tests for carcinogens: Report of the International Programme on Chemical Safety´s Collaborative Study on in vivo assays.

[id_DUK_614] Samet Jonathan M, Chiu Weihsueh A, Cogliano Vincent, Jinot Jennifer, Kriebel David, Lunn Ruth M, Beland Frederick A, Bero Lisa, Browne Patience, Fritschi Lin, Kanno Jun, Lachenmeier Dirk W, Lan Qing, Lasfargues Gérard, Le Curieux Frank, Peters Susan, Shubat Pamela, Sone Hideko, White Mary C, Williamson Jon, Yakubovskaya Marianna, Siemiatycki Jack, White Paul A, Guyton Kathryn Z, Schubauer-Berigan Mary K, Hall Amy L, Grosse Yann, Bouvard Véronique, Benbrahim-Tallaa Lamia, El Ghissassi Fatiha, Lauby-Secretan Béatrice, Armstrong Bruce, Saracci Rodolfo, Zavadil Jiri, Straif Kurt, Wild Christopher P (2020). The IARC monographs: updated procedures for modern and transparent evidence synthesis in cancer hazard identification. J Natl Cancer Inst.

[id_DUK_615] Serota D. G., Hoberman A. M., Friedman M. A., Gad S. C. (1988). Three-generation reproduction study with caprolactam in rats. J Appl Toxicol.

[id_DUK_616] Sheldon T. (1989). An evaluation of caprolactam and benzoin in the mouse micronucleus test. Mutat Res.

[id_DUK_617] Sheldon T. (1989). Chromosomal damage induced by caprolactam in human lymphocytes. Mutat Res.

[id_DUK_618] Smith Carr J, Perfetti Thomas A (2019). An approximated one-quarter of IARC Group 3 (unclassifiable) chemicals fit more appropriately into IARC Group 4 (probably not carcinogenic). Toxicol Res Appl.

[id_DUK_619] Sterner A. (2012). Tranexamsäure versus ε-Aminocapronsäure bei kinderherzchirurgischen Eingriffen.

[id_DUK_620] Swaen Gerard M. H., Scheffers Theo, de Cock Johan, Slangen Jos, Drooge Hinkelien (2005). Leukemia risk in caprolactam workers exposed to benzene. Ann Epidemiol.

[id_DUK_621] Tibaldi Rosalie, ten Berge Wil, Drolet Daniel (2014). Dermal absorption of chemicals: estimation by IH SkinPerm. J Occup Environ Hyg.

[id_DUK_622] Triebig Gerhard, Triebig-Heller Isabel, Bruckner Thomas (2016). Exposure study to examine chemosensory effects of ɛ-caprolactam in healthy men and women. Inhal Toxicol.

[id_DUK_623] Unger P. D., Salerno A. J., Friedman M. A. (1981). Disposition of [^14^C]caprolactam in the rat. Food Cosmet Toxicol.

[id_DUK_624] Vogel E. W. (1989). Caprolactam induces genetic alterations in early germ cell stages and in somatic tissue of D. melanogaster. Mutat Res.

[id_DUK_625] Waddell W. J., Marlowe C., Friedman M. A. (1984). The distribution of [^14^C]caprolactam in male, female and pregnant mice. Food Chem Toxicol.

[id_DUK_626] Wiench K., Bartsch R., Leibold Edgar, Jahnke Gunnar, Hartwig A., MAK Commission (2016). Methacrylsäure. MAK Value Documentation in German language. MAK Collect Occup Health Saf.

[id_DUK_627] Wild D., Baß R, Glocklin V, Grosdanoff P, Henschler D, Kilbey B, Müller D, Neubert D (1984). Critical evaluation of mutagenicity tests.

[id_DUK_628] Wolkoff Peder (2018). Indoor air humidity, air quality, and health - An overview. Int J Hyg Environ Health.

[id_DUK_629] Wolkoff P., Nøjgaard J. K., Franck C., Skov P. (2006). The modern office environment desiccates the eyes?. Indoor Air.

[id_DUK_630] Working P. K. (1989). Assessment of unscheduled DNA synthesis in Fischer 344 rat pachytene spermatocytes exposed to caprolactam or benzoin in vivo. Mutat Res.

[id_DUK_631] Ziegler A. E., Zimmer H., Triebig G. (2008). Exposure study on chemosensory effects of ε-caprolactam in the low concentration range. Int Arch Occup Environ Health.

